# Radiolabeled Coordination Polymer‐Loaded Microneedles for Synergistic Melanoma Brachytherapy–Immunotherapy via STING Activation and Pyroptosis

**DOI:** 10.1002/EXP.20250737

**Published:** 2026-06-22

**Authors:** Pian Yu, Shijun Xiang, Lu Hao, Jessica C. Hsu, Kaixuan Li, Rongxuan Yan, Ming Zhou, Yongxiang Tang, Ying Peng, Weibo Cai, Cong Peng, Peng Liu, Shuo Hu

**Affiliations:** ^1^ Department of Nuclear Medicine Xiangya Hospital Central South University Changsha China; ^2^ The Department of Dermatology Xiangya Hospital Central South University Changsha China; ^3^ Hunan Key Laboratory of Skin Cancer and Psoriasis Hunan Engineering Research Center of Skin Health and Disease Xiangya Hospital Changsha China; ^4^ Furong Labratory Changsha China; ^5^ National Engineering Research Center of Personalized Diagnostic and Therapeutic Technology Changsha China; ^6^ National Clinical Research Center for Geriatric Disorders Xiangya Hospital Changsha China; ^7^ Departments of Radiology and Medical Physics University of Wisconsin‐Madison Madison Wisconsin USA; ^8^ Xiangya School of Pharmaceutical Sciences Central South University Changsha China; ^9^ Key Laboratory of Biological Nanotechnology Changsha China

**Keywords:** melanoma, STING pathway, pyroptosis, radiolabeled coordination polymers, microneedles

## Abstract

Melanoma remains a highly aggressive malignancy with limited response to current immunotherapies due to its immunosuppressive tumor microenvironment. To overcome this limitation, we developed a radiolabeled coordination polymer, ^177^Lu‐GAMP, through the self‐assembly of ^177^Lu^3+^ with adenosine monophosphate (AMP) and guanosine monophosphate, exhibiting coordination‐feature resemblance to the endogenous STING agonist cGAMP, thereby enabling activation of the STING pathway. We further incorporated ^177^Lu‐GAMP into a dissolvable microneedle patch (^177^Lu‐GAMP@MN) for localized, minimally invasive delivery to melanoma lesions. Our results demonstrate that ^177^Lu‐GAMP@MN effectively penetrated the skin and retained at the tumor site, leading to robust STING activation and Gasdermin E‐mediated pyroptosis. This, in turn, promoted dendritic cell maturation and enhanced T cell infiltration. In vivo, ^177^Lu‐GAMP@MN significantly suppressed subcutaneous melanoma growth, prolonged survival, and elicited strong antitumor immune responses. When combined with anti‐PD‐L1 monoclonal antibodies, the treatment achieved synergistic tumor regression, improved effector T cell function, and induced durable immunological memory, demonstrating significant inhibition of both primary and distant tumors in murine models. Collectively, this work presents a transdermal brachytherapeutic‐immunomodulatory strategy for melanoma treatment, offering promising potential for enhanced antitumor immunotherapy.

## Graphical abstract

We developed a dissolvable microneedle patch that locally delivers a radiolabeled coordination polymer designed to simultaneously destroy tumor cells and stimulate antitumor immunity through STING activation and pyroptosis. This strategy significantly inhibited melanoma growth and improved the effectiveness of immune checkpoint therapy, highlighting a promising minimally invasive platform for cancer treatment.

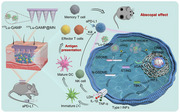

## 1 Introduction

Melanoma is a highly aggressive skin cancer characterized by rapid progression, early metastasis, and frequent recurrence [1]. In recent decades, both the incidence and mortality of melanoma have risen sharply, with approximately 160,000 new cases and 48,000 deaths reported worldwide each year [2]. Standard treatments such as surgical resection and chemotherapy often result in poor long‐term outcomes, substantial side effects, and the development of drug resistance [3, 4]. Incomplete surgical resection occurs in 0.4% to 35.7% of cases, often leading to recurrence or metastasis that accounts for the majority of melanoma‐related mortality [5, 6]. Once recurrent or metastatic, the disease is largely incurable, with 5‐year survival rates ranging from 9% to 28% [7]. Although combination chemotherapy regimens can improve response rates, they fail to significantly improve overall survival, underscoring their limited efficacy [8]. Immunotherapy has revolutionized melanoma management and brought new hope for patients. Among these therapies, immune checkpoint inhibitors (ICIs) are the most widely used. By blocking immune‐checkpoint targets such as PD‐1/PD‐L1 and CTLA‐4 on tumor or immune cells, ICIs restore antitumor immunity and enhance T cell responses [9]. However, clinical trials have shown variable efficacy, with overall response rates remaining below 40% [10, 11]. One of the primary reasons for this limited efficacy is the immunosuppressive tumor microenvironment (TME), which is marked by: 1) rapid tumor cell proliferation and low intrinsic immunogenicity; 2) poor immune cell infiltration; and 3) TME factors that impair immune cell function [12, 13, 14, 15]. Therefore, strategies that promote immune cell activation and infiltration into the TME are crucial to improving immunotherapy outcomes in melanoma [16, 17].

The cyclic GMP‐AMP synthase (cGAS)‐stimulator of interferon genes (STING) pathway is a key component of innate immunity [18]. It allows mammalian cells to detect aberrant double‐stranded DNA (dsDNA) from pathogens or tumor cells, and to initiate immune responses via the cGAS‐STING axis [19]. Upon binding to dsDNA, cGAS catalyzes the synthesis of 2′‐3′ cyclic GMP‐AMP (cGAMP), which serves as a second messenger to bind and activate STING dimers on the endoplasmic reticulum. Activated STING recruits TANK‐binding kinase 1 (TBK1) and phosphorylates interferon regulatory factor 3 (IRF3), leading to the production of type I interferons (IFNs) [20]. When combating malignant tissues, type I IFN acts as a bridge between innate and adaptive immunity, thereby activating natural killer (NK) cells and dendritic cells (DCs), enhancing antigen presentation, and promoting T‐cell recruitment and infiltration into the TME [21]. Thus, effective STING pathway activation represents a promising strategy for enhancing antitumor immunity.

Recent efforts have focused on developing STING agonists. Given that cGAMP directly stimulates STING pathway activation, most STING agonists are structurally derived from cGAMP, including cyclic dinucleotides (CDNs) and their synthetic analogs [22]. Preclinical studies have demonstrated that these agents can improve T‐cell responses and increase tumor immunogenicity in melanoma [23, 24]. However, clinical translation has been hampered by several limitations: STING agonists are typically administered via intratumoral injection, have poor systemic bioavailability, are rapidly degraded by phosphodiesterases, and suffer from poor membrane permeability due to their high polarity [25, 26]. To overcome these barriers, there is growing interest in designing STING agonists with improved stability and cellular uptake. Metal‐organic coordination polymers (CPs), formed through the coordination of metal ions and organic ligands, offer a versatile platform for drug design [27, 28]. Notably, we discovered that CPs assembled from Lu^3+^ and nucleotide ligands such as adenosine monophosphate (AMP) and guanosine monophosphate (GMP) exhibit coordination‐feature resemblance to cGAMP. This suggests that the Lu‐GAMP complex could function as a potent STING agonist for cancer immunotherapy.

Radiotherapy (RT) remains a cornerstone of cancer treatment because it directly destroys tumor cells while also modulating the immune landscape [29, 30]. Nevertheless, conventional external‐beam RT suffers from limited dose precision and off‐target toxicity [31]. Brachytherapy (BT), an alternative approach involving localized implantation of α‐ or β‐emitting radionuclides, offers greater safety and efficacy by delivering high‐linear energy transfer (LET) radiation directly to tumor sites [32, 33]. Among available radionuclides, lutetium‐177 (^177^Lu) stands out due to its clinically favorable properties, including a half‐life of 6.7 days, tissue penetration of 0.67 mm, and β‐emission energy of 0.498 MeV, making it ideal for treating superficial tumors while sparing healthy tissue [34]. We found that ^177^Lu can induce Gasdermin E (GSDME)‐mediated pyroptosis, a form of inflammatory cell death characterized by the release of immunostimulatory molecules that amplify antitumor immunity [35, 36]. However, the immunomodulatory effects of BT remain underexplored.

Transdermal delivery has emerged as a promising route for immunotherapy, particularly in melanoma [37]. The skin is rich in professional antigen‐presenting cells and plays a critical role in immune surveillance [38]. Delivering immune modulators through the skin can efficiently activate local immune responses at low doses, reduce systemic side effects, and establish lasting immune memory to prevent metastasis [39]. Among transdermal platforms, dissolving microneedles (DMNs) have gained attention as a minimally invasive and effective delivery system [40, 41, 42, 43]. Given that melanoma primarily originates in the epidermis, DMNs can painlessly penetrate the stratum corneum to deliver therapeutics directly into the tumor site, while also enhancing patient compliance [44].

**SCHEME 1:**
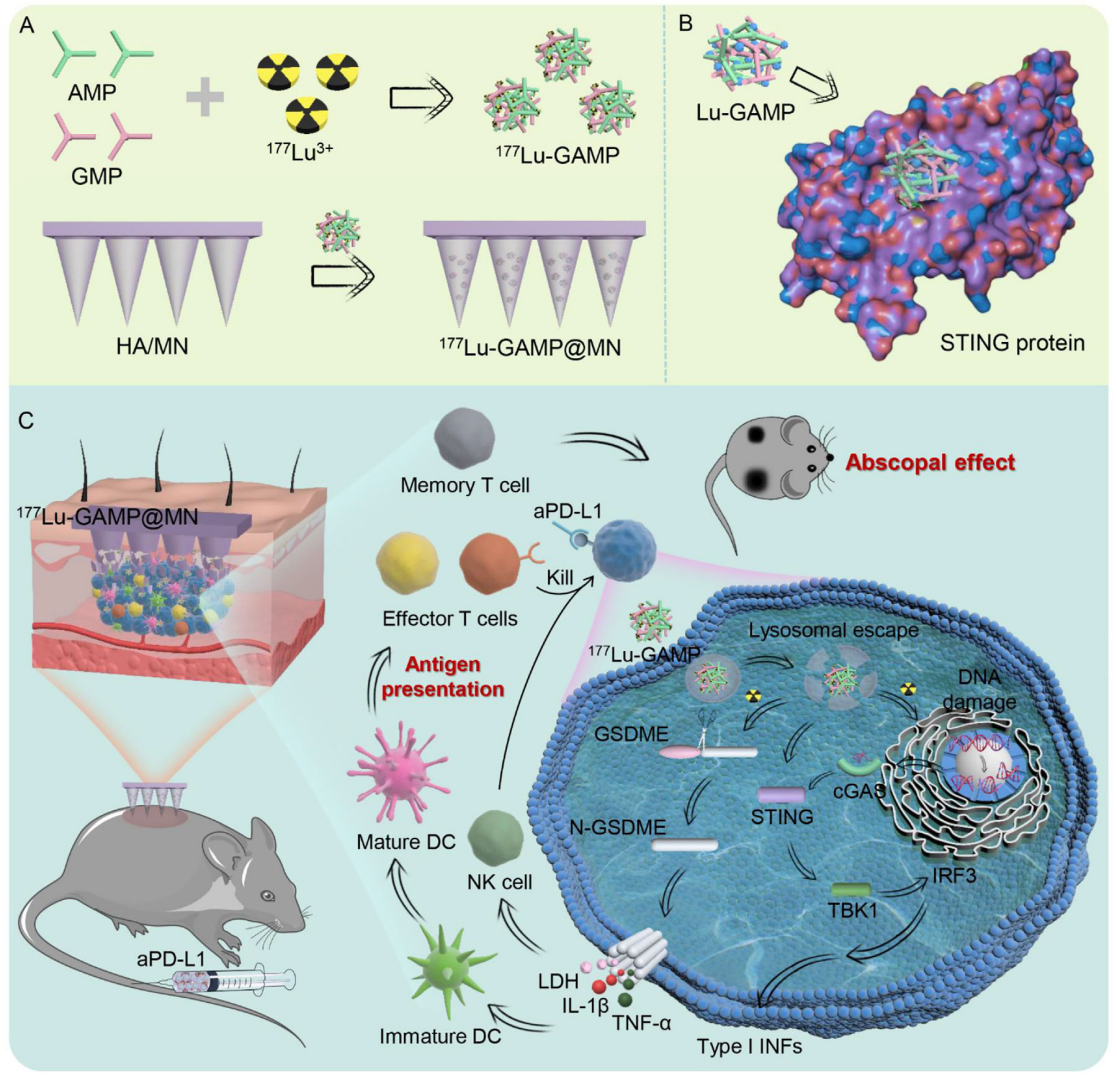
(A) Schematic illustration of the fabrication process for the radiolabeled CPs‐loaded microneedle platform (^177^Lu‐GAMP@MN). (B) Lu‐GAMP exhibits strong binding affinity to the STING protein. (C) Conceptual diagram showing enhanced melanoma immunotherapy modulated by ^177^Lu‐GAMP@MN through STING pathway activation and GSDME‐mediated pyroptosis.

Herein, we designed a novel radiolabeled CPs, namely ^177^Lu‐GAMP, by self‐assembling ^177^Lu^3+^ with AMP and GMP. We then incorporated ^177^Lu‐GAMP into a dissolvable microneedle patch (^177^Lu‐GAMP@MN) for transdermal delivery to melanoma lesions. Upon application, the microneedles dissolve, releasing ^177^Lu‐GAMP, which is readily internalized by tumor cells. This triggers robust STING pathway activation and GSDME‐mediated pyroptosis, leading to the release of inflammatory cytokines and type I IFNs that promote immune cell recruitment. Furthermore, when combined with systemic administration of anti‐PD‐L1 monoclonal antibodies (aPD‐L1), this approach elicits synergistic antitumor immune responses and durable immunological memory, suppressing both primary and metastatic melanoma in murine models (Scheme 1).

## 2 Results and Discussion

### 2.1 Preparation of Lu‐GAMP for Intracellular STING Pathway Activation

**FIGURE 1:**
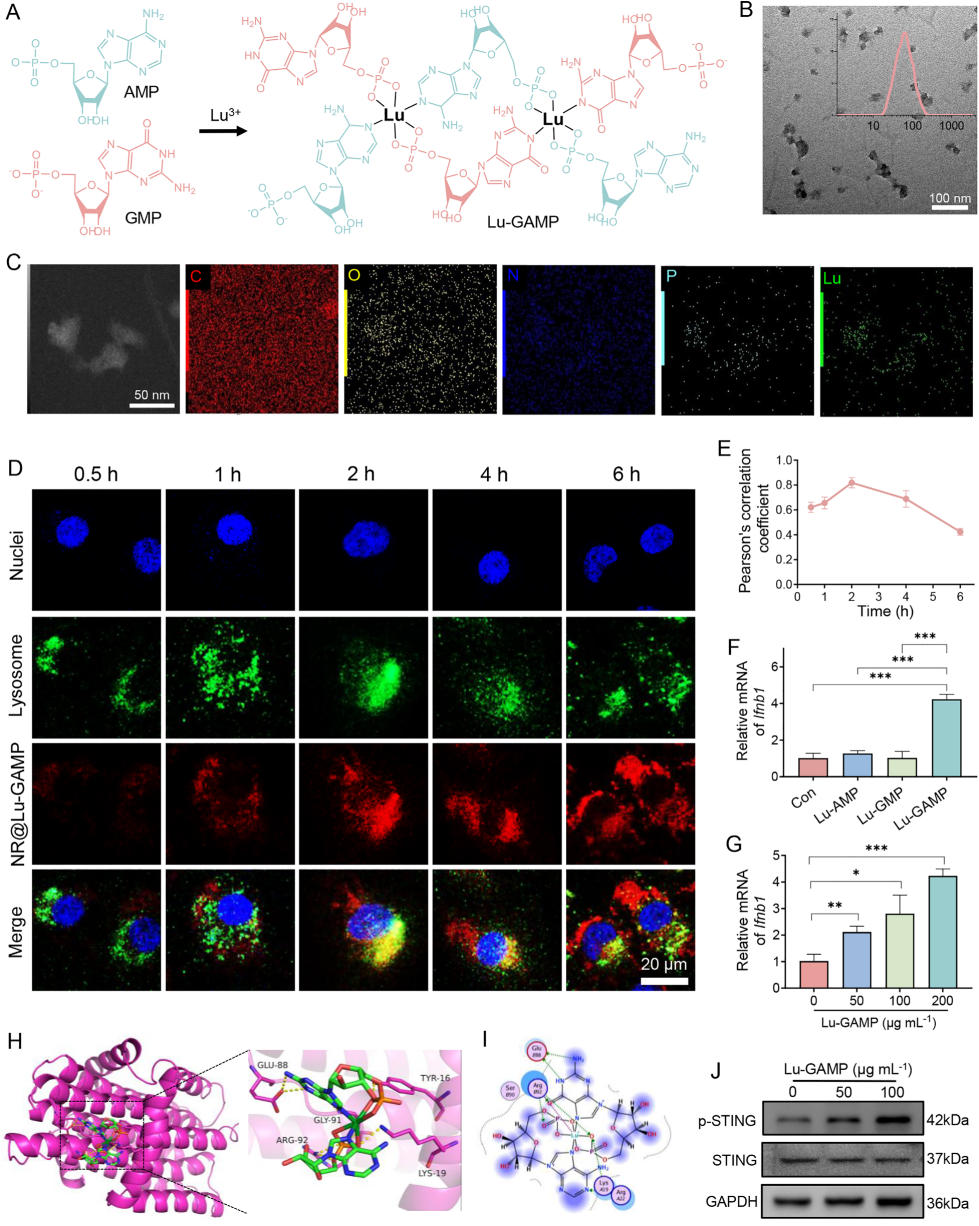
(A) Schematic illustration of the self‐assembly of Lu^3+^ with AMP and GMP to form Lu‐GAMP coordination polymers. (B) TEM image and DLS analysis of Lu‐GAMP. (C) Elemental mapping images of Lu‐GAMP. (D) Colocalization observation by CLSM and (E) Pearson's correlation coefficients of NR‐labeled Lu‐GAMP (red) and Lysotracker (green) (*n* = 3). Expression of Ifnb1 in B16‐F10 cells treated with (F) various CPs and (G) with different concentrations of Lu‐GAMP (*n* = 3). (H) 3D and (I) 2D binding modes of Lu‐GAMP within the active site of mSTING (4KC0). (J) Western blot analysis of STING and p‐STING in B16‐F10 cells treated with increasing concentrations of Lu‐GAMP. Data are presented as the mean ± SD. *p < 0.05, **p < 0.01, ***p < 0.001.

The self‐assembly of Lu^3+^ with AMP and GMP in aqueous solution resulted in the formation of opalescent precipitates, which were subsequently washed and sonicated to yield nanoscale coordination polymers, referred to as Lu‐GAMP (Figure 1A). Transmission electron microscopy (TEM) and dynamic light scattering (DLS) analysis revealed that Lu‐GAMP exhibited an irregular morphology with an average diameter of 67.3 nm (Figure 1B and Table S1). Elemental mappings by energy‐dispersive X‐ray spectroscopy (EDX) confirmed the presence of C, N, O, P, and Lu, indicating a homogeneous distribution of Lu^3+^ ions within the Lu‐GAMP structure (Figure 1C and Table S2). Furthermore, Lu‐GAMP demonstrated excellent stability in both PBS buffer and complete Dulbecco's modified Eagle's medium (DMEM) supplemented with fetal bovine serum (FBS) (Figure S1A and S1B), as well as maintained stability in aqueous solution over extended periods (Figure S1C).

To assess cellular uptake and intracellular trafficking, B16‐F10 melanoma cells were incubated with Nile red (NR)‐labeled Lu‐GAMP. First, the NR‐labeling stability was measured at different time intervals following centrifugation. After 24 h of incubation in medium containing 10% FBS, NR‐labeled Lu‐GAMP exhibited less than 10% NR leakage, confirming the stability of the labeling (Figure S2). Furthermore, cellular uptake and lysosomal escape was investigated by co‐staining with endo/lysosomal trackers. A time‐dependent increase in intracellular red fluorescence was observed, peaking at 6 h, indicating efficient internalization of the nanoparticles (Figure 1D and Figure S3). Pearson's correlation coefficients between Lu‐GAMP and lysosomal markers were calculated to quantify escape efficiency. At early time points (0–2 h), Lu‐GAMP co‐localized strongly with lysosomes (correlation coefficient ∼0.82), however, by 6 h, the coefficient decreased to 0.42, indicating successful lysosomal escape, a prerequisite for effective STING pathway activation (Figure 1E).

To elucidate the role of the AMP and GMP components, control Lu‐AMP and Lu‐GMP coordination polymers were synthesized (Table S1) and assessed for their ability to stimulate a type I IFN response in B16‐F10 cells via STING signaling. The qPCR analysis showed that only Lu‐GAMP significantly upregulated type I IFN‐related gene expression, including Ifnb1, in a concentration‐dependent manner after 24 h incubation (Figure 1F, G). This trend was further confirmed at the protein level by ELISA, where Lu‐GAMP induced a marked and dose‐dependent increase in IFN‐β secretion. In contrast, Lu‐AMP and Lu‐GMP failed to elicit detectable IFN responses at both mRNA and protein levels, even at concentrations up to 200 µg mL^−1^ (Figure S4). To verify the activation of STING signaling pathway by Lu‐GAMP, molecular docking and molecular dynamics (MD) simulations were performed. Docking results showed that Lu‐GAMP binds to the active site of mouse STING (mSTING) with a binding energy of −7.82 kcal mol^−1^, forming hydrogen bonds with key residues (Glu^88^, Tyr^16^, Gly^91^, Arg^92^, and Lys^19^) (Figure 1H,I). MD simulations showed that the root mean square deviation (RMSD) profiles of both the protein alone and the protein‐ligand complex remained stable, indicating that the systems reached equilibrium during the simulation (cutoff = 0.2) (Figure S5). Binding free energy analysis further confirmed the formation of a stable complex (Table S3), with van der Waals and electrostatic interactions as the main driving forces, while polar solvation energy acted as the primary unfavorable contribution (Figure S6). Similar interactions were observed with human STING (hSTING) (Figure S7). Western blot analysis further confirmed the upregulation of phosphorylated STING (p‐STING) in B16‐F10 cells treated with Lu‐GAMP, with levels increasing in a dose‐dependent manner (Figure 1J and Figure S8), supporting the activation of the STING signaling cascade.

### 2.2 ^177^Lu Induces GSDME‐Mediated Pyroptosis in Melanoma Cells

**FIGURE 2:**
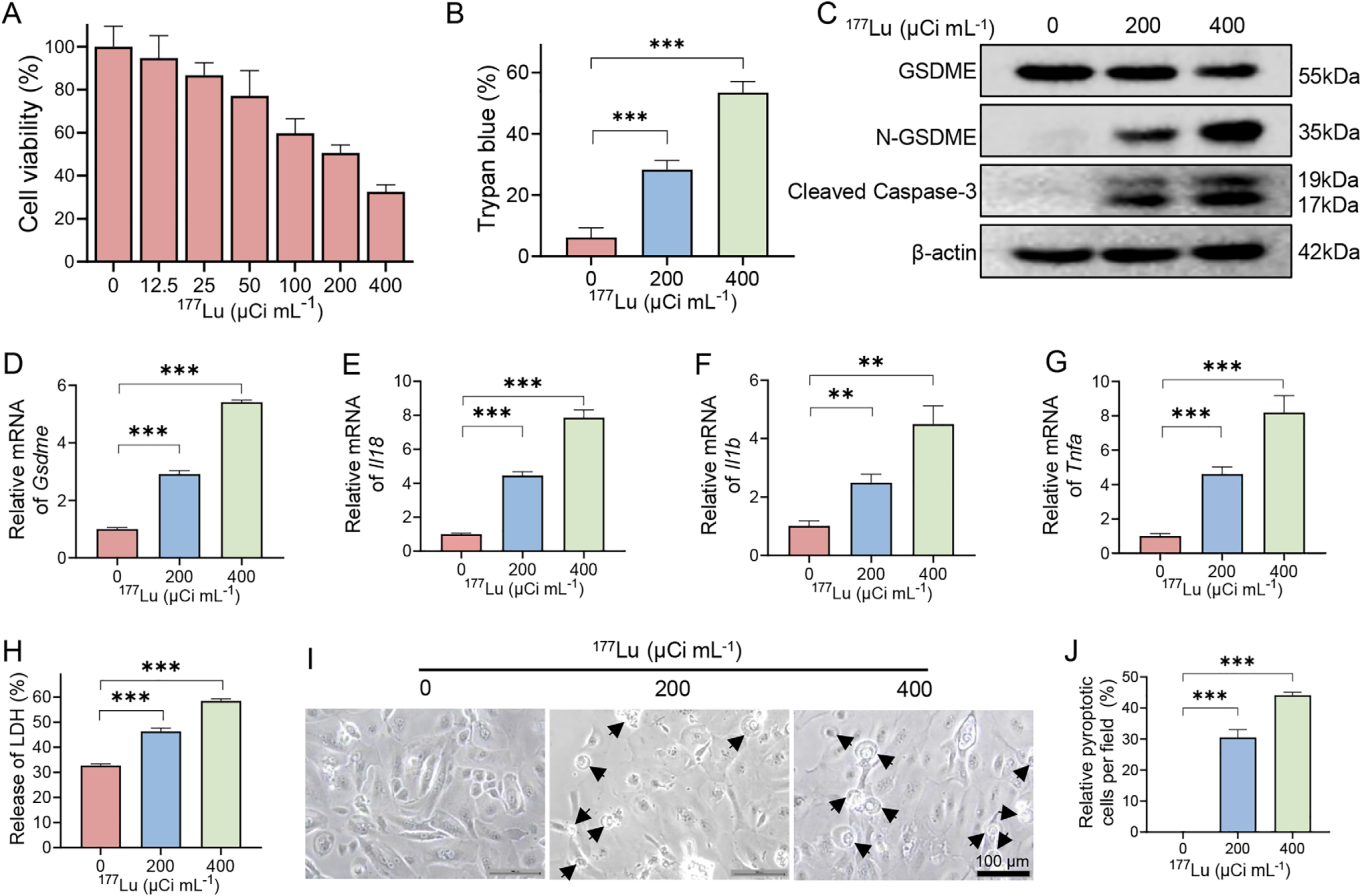
(A) In vitro cell viability (*n* = 5) and (B) percentage of trypan blue‐positive B16‐F10 cells after treatment with varying concentrations of ^177^Lu (*n* = 3). (C) Western blot analysis of GSDME, N‐GSDME, and cleaved Caspase‐3 protein expression in B16‐F10 cells following ^177^Lu treatment. RT‐qPCR detection of pyroptosis‐related gene expression, including (D) *Gsdme*, (E) *Il‐18*, (F) *Il‐1b*, and (G) *Tnfa*. (H) LDH release in the supernatant after treatment with 200 and 400 µCi mL^−1 177^Lu (*n* = 3). (I) Bright‐field microscopy images showing morphological features of pyroptotic cells, and (J) quantification of pyroptotic cell ratio (*n* = 3). Data are presented as the mean ± SD. *p < 0.05, **p < 0.01, ***p < 0.001.

BT, a form of internal radiotherapy, delivers localized tumor ablation through implanted radionuclide sources and is widely employed in the treatment of various solid tumors [45]. However, the mechanisms of tumor cell death induced by radionuclides, particularly those involving immune‐related pathways, remain poorly understood and are insufficiently characterized. Here, we investigated the mechanism of cell death induced by the therapeutic radionuclide ^177^Lu in melanoma cells. The cytotoxic effect of ^177^Lu on B16‐F10 cells was first assessed using the CCK‐8 assay. As expected, ^177^Lu treatment significantly reduced B16‐F10 cell viability in a dose‐dependent manner (Figure 2A). Furthermore, trypan blue staining revealed a marked increase in membrane‐compromised (trypan blue‐positive) cells following ^177^Lu exposure (Figure 2B), indicating elevated membrane permeability and increased cell death. Given previous evidence suggesting that radiopharmaceuticals can induce pyroptosis, we hypothesized that ^177^Lu might trigger this form of pro‐inflammatory cell death in melanoma cells [46]. To test this, we examined the cleavage of GSDME, a defining event in pyroptosis. Western blot analysis revealed a dose‐dependent increase in the cleaved N‐terminal fragment of GSDME (GSDME‐N) following ^177^Lu treatment, indicating GSDME activation. Additionally, we observed a corresponding dose‐dependent increase in the cleaved, activated form of Caspase‐3, confirming the induction of Caspase‐3/GSDME‐mediated pyroptosis signaling (Figure 2C and Figure S9). Consistent with this, ^177^Lu also upregulated the mRNA expression of key pyroptosis‐related genes, including *Gsdme* and the pro‐inflammatory cytokines *Il‐18*, *Il‐1b*, and *Tnfa* (Figure 2D–G). Importantly, ^177^Lu elicited significant lactate dehydrogenase (LDH) release from B16‐F10 cells (Figure 2H), a direct indicator of pyroptotic membrane rupture. Phase‐contrast microscopy further revealed characteristic pyroptotic morphology, including extensive cellular swelling and membrane blebbing (indicated by black arrows in Figure 2I,J). Collectively, these results demonstrated that ^177^Lu induces GSDME‐mediated pyroptosis in murine melanoma B16‐F10 cells, uncovering an immune‐relevant mechanism of cell death that may contribute to the immunomodulatory effects of radionuclide‐based therapy.

### 2.3 ^177^Lu‐GAMP Induces Tumor Cell Death and DCs Maturation via STING Activation and GSDME‐Mediated Pyroptosis

**FIGURE 3:**
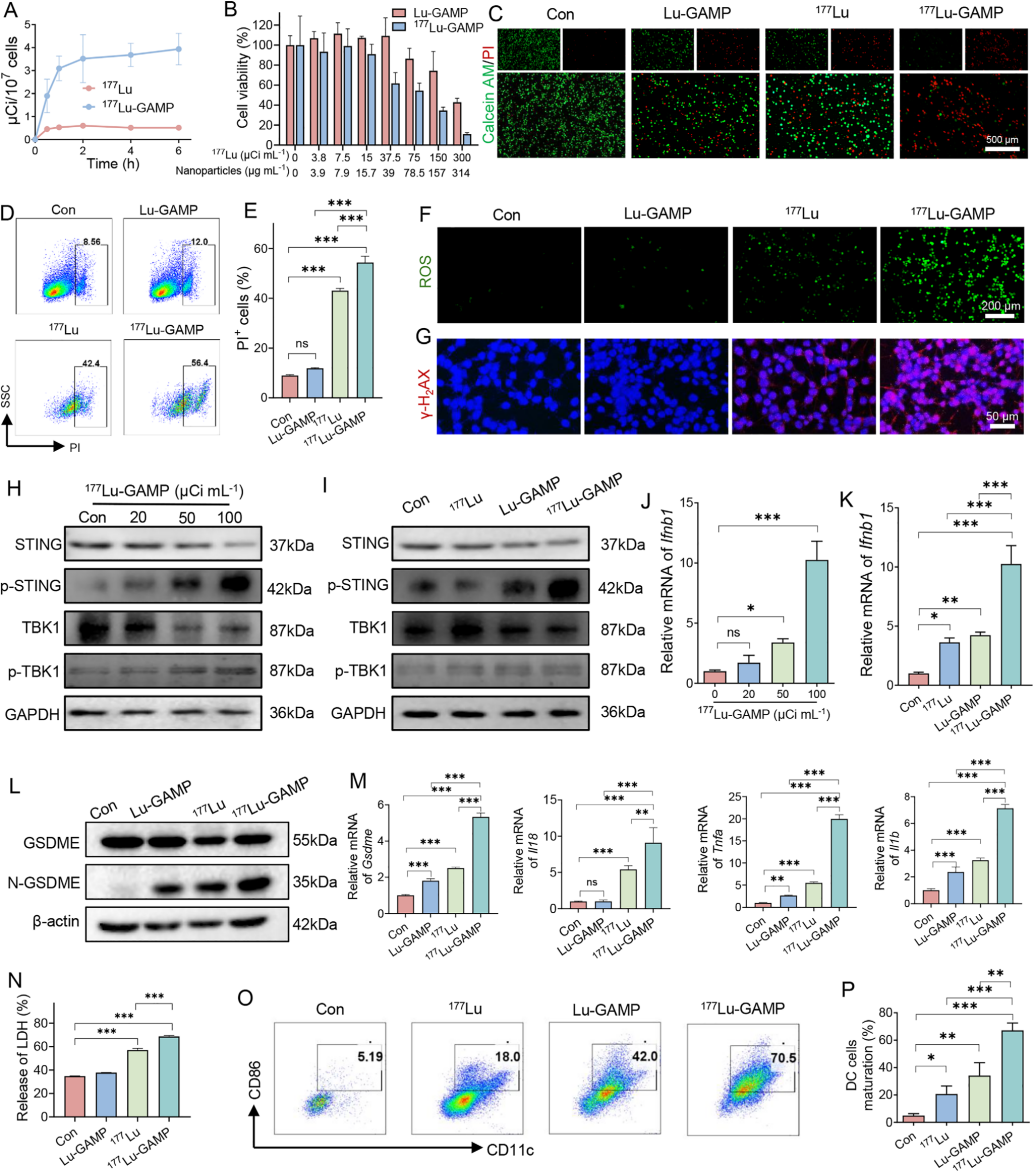
(A) Cellular uptake of free ^177^Lu and ^177^Lu‐GAMP by B16‐F10 cells (*n* = 4). (B) Viability of B16‐F10 cells incubated with various concentrations of Lu‐GAMP and ^177^Lu‐GAMP (*n* = 5). (C) Fluorescence images of live/dead staining under different treatment conditions. Evaluation of cell apoptosis and necrosis by (D) flow cytometry and (E) corresponding quantification (*n* = 3). (F) Fluorescence imaging of ROS generation in B16‐F10 cells. (G) Immunofluorescence staining of γ‐H_2_AX expression. Western blot analysis of STING, p‐STING, TBK1, and p‐TBK1 following treatment with (H) increasing concentrations of ^177^Lu‐GAMP, and (I) under various therapeutic conditions (^177^Lu: 100 µCi mL^−1^, Lu‐GAMP: 100 µg mL^−1^). RT‐qPCR analysis of *Ifnb1* mRNA levels after treatment with (J) graded doses of ^177^Lu‐GAMP and (K) various formulations (*n* = 3). (L) Western blot analysis of GSDME and N‐GSDME expression following different treatments (^177^Lu: 100 µCi mL^−1^, Lu‐GAMP: 100 µg mL^−1^). (M) RT‐qPCR analysis of pyroptosis‐related genes including *Gsdme*, *Il‐18*, *Il‐1b*, and *Tnfa* (*n* = 4). (N) LDH release in culture supernatants post‐treatment (*n* = 3). (O) Flow cytometry plots and (P) quantification of CD11c^+^CD86^+^ mature dendritic cells in DC2.4 cells treated with different formulations (*n* = 3). Data are presented as the mean ± SD. ns, not significant (*p* > 0.05), *p < 0.05, **p < 0.01, ***p < 0.001.

Subsequently, ^177^Lu‐GAMP was synthesized by radiolabeling Lu‐GAMP with a trace amount of [^177^Lu]LuCl_3_ using the same procedure employed for Lu‐GAMP preparation [47]. After removal of free ^177^Lu by centrifugation, ^177^Lu‐GAMP with a radiolabeling rate exceeding 90% was obtained. The resulting product showed excellent radiolabeling stability in PBS, DMEM containing 10% FBS or 10% mouse serum (Figure S10). ^177^Lu‐GAMP exhibited time‐dependent uptake in B16‐F10 melanoma cells, reaching an intracellular accumulation of 3 µCi per 10^7^ cells after 1 h of incubation (Figure 3A). In comparison, cells treated with free ^177^Lu displayed significantly lower radioactivity, underscoring the enhanced internalization efficiency of the coordination polymer formulation. Given its efficient cellular uptake, the cytotoxic effects of ^177^Lu‐GAMP were subsequently evaluated using the CCK‐8 assay. Both ^177^Lu‐GAMP and Lu‐GAMP induced dose‐dependent cytotoxicity, with ^177^Lu‐GAMP demonstrating greater toxicity due to the radiotherapeutic contribution of ^177^Lu (Figure 3B). To visualize cell viability, Calcein AM (green) and propidium iodide (PI, red) dual‐fluorescence staining was conducted. While control cells displayed strong green fluorescence, indicative of intact membranes and high viability (Figure 3C and Figure S11), cells treated with Lu‐GAMP, ^177^Lu, or ^177^Lu‐GAMP showed increasing red fluorescence, with the latter exhibiting the most intense PI signal, reflecting pronounced cell death. Flow cytometry further confirmed a markedly higher percentage of PI‐positive cells in the ^177^Lu‐GAMP group relative to all other treatment groups (Figure 3D,E), in agreement with the CCK‐8 results. To explore the underlying mechanisms of cytotoxicity, we examined intracellular reactive oxygen species (ROS) levels using the fluorogenic probe DCFH‐DA. Cells treated with ^177^Lu‐GAMP exhibited strong green fluorescence, indicating elevated ROS generation (Figure 3F and Figure S12). Additionally, immunofluorescence staining of γ‐H_2_AX revealed extensive DNA double‐strand breaks in ^177^Lu‐GAMP‐treated cells (Figure 3G and Figure S13). Together, these results suggest that ^177^Lu‐GAMP elicits synergistic cytotoxic effects through enhanced ROS production and DNA damage in melanoma cells.

To assess the activation of the STING signaling pathway, Western blot analysis was performed to detect the expression of p‐STING and phosphorylated TBK1 (p‐TBK1). A dose‐dependent increase in p‐STING and p‐TBK1 expression was observed in ^177^Lu‐GAMP‐treated B16‐F10 cells, exceeding levels seen in the ^177^Lu and Lu‐GAMP groups (Figure 3H,I and Figures S14 and S15). Upregulation of *Ifnb* mRNA further confirmed STING pathway activation following ^177^Lu‐GAMP treatment (Figure 3J,K). To elucidate the role of cGAS in STING activation, a cGAS inhibitor (RU.521) was employed. Inhibition of cGAS markedly reduced STING pathway activation induced by ^177^Lu, indicating a cGAS‐dependent mechanism likely associated with radiation‐induced DNA damage (Figure S16A and S16B). In contrast, Lu‐GAMP retained its ability to activate STING under cGAS inhibition, supporting a direct STING agonistic effect independent of cGAS (Figure S16C and S16D). In addition, the immunogenic nature of ^177^Lu‐GAMP was supported by its ability to induce GSDME‐mediated pyroptosis. Compared with ^177^Lu monotherapy, ^177^Lu‐GAMP treatment led to increased GSDME cleavage (Figure 3L and Figure S17) and elevated transcription of pyroptosis‐related cytokines, including *Gsdme, Il‐18*, *Il‐1b*, and *Tnfa* (Figure 3M). This lytic form of cell death was also associated with elevated LDH release (Figure 3N). Critically, to distinguish specific pyroptotic lysis from non‐specific membrane damage, GSDME expression was knocked down using siRNA. GSDME knockdown (Figure S18A) specifically and significantly attenuated the ^177^Lu‐GAMP‐induced LDH release (Figure S18B), confirming the pyroptosis‐dependency of this effect. We then examined the function of ^177^Lu‐GAMP to induce immunogenic cell death (ICD). Compared to the control, both ^177^Lu and ^177^Lu‐GAMP significantly induced CRT exposure, as well as the release of HMGB1 and ATP, among which ^177^Lu‐GAMP exhibited the most pronounced effect. (Figure S19). Finally, to examine the downstream immunological effects, DC maturation was analyzed by flow cytometry. Treatment with ^177^Lu‐GAMP significantly increased the population of CD11c^+^CD86^+^ mature DCs (Figure 3O,P), indicating enhanced antigen‐presenting function. This phenotypic change coincided with elevated secretion of damage‐associated molecular patterns (DAMPs), suggesting that ^177^Lu‐GAMP‐mediated ICD effectively promotes DC activation. Collectively, these results demonstrate that ^177^Lu‐GAMP exerts dual immunomodulatory effects associated with both activation the STING signaling pathway and induction of GSDME‐mediated pyroptosis.

### 2.4 Fabrication of ^177^Lu‐GAMP@MN Microneedle for Subcutaneous Melanoma Application

**FIGURE 4:**
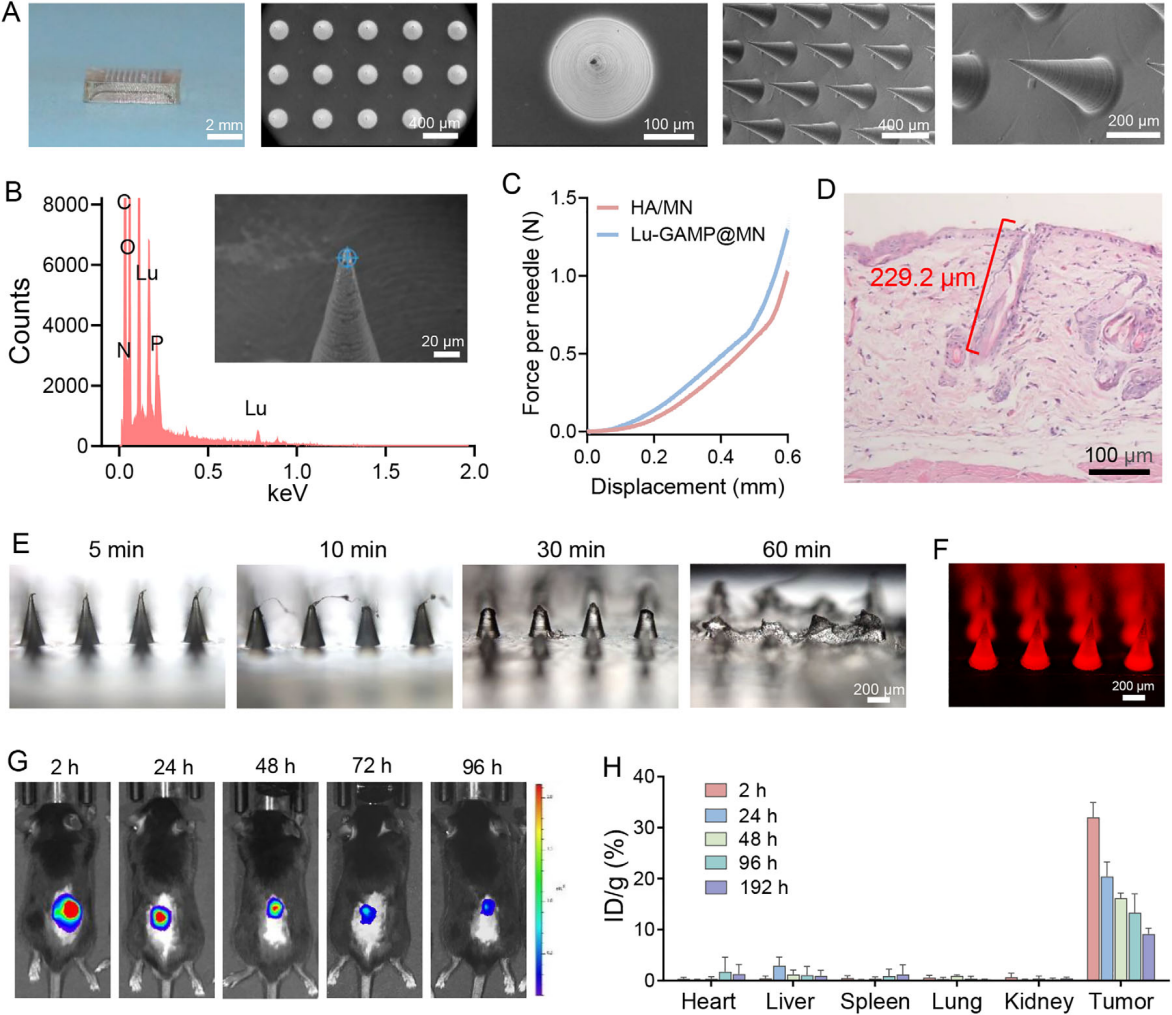
(A) Photograph, optical microscopy and SEM images of Lu‐GAMP@MN. (B) SEM‐EDS analysis of the needle tip of Lu‐GAMP@MN. (C) Mechanical compression strength comparison between HA/MN and Lu‐GAMP@MN. (D) H&E staining of murine skin following application of Lu‐GAMP@MN. (E) Dissolution profile of Lu‐GAMP@MN after insertion into murine skin. (F) Fluorescence image of NR‐labeled Lu‐GAMP‐integrated MNs. (G) In vivo fluorescence imaging of NR‐labeled Lu‐GAMP@MN in subcutaneous B16‐F10‐tumor‐bearing mice. (H) In vivo biodistribution of radioactive ^177^Lu‐GAMP@MN at various time points following skin insertion (*n* = 3).

Microneedle (MN) technology offers a minimally invasive strategy for transdermal drug delivery by creating microscopic pores in the skin, providing an efficient alternative for treating superficial tumors. We then fabricated ^177^Lu‐GAMP@MN by incorporating ^177^Lu‐GAMP into hyaluronic acid (HA)‐based microneedles using a micro‐molding technique. Briefly, ^177^Lu‐GAMP was homogeneously dispersed in a HA pre‐gel solution, which was then cast into microneedle molds. After drying, the solidified microneedles were demolded to yield the final ^177^Lu‐GAMP@MN formulation. Optical and scanning electron microscopy (SEM) confirmed the 3D conical architecture of Lu‐GAMP@MN, with uniform shape and consistent dimensions (Figure 4A). SEM‐EDS analysis revealed the homogenous distribution of C, O, N, P, and Lu elements within the needle tips, validating the successful incorporation of Lu‐GAMP (Figure 4B). Mechanical strength testing showed smooth and continuous force‐displacement curves for both blank HA microneedles and Lu‐GAMP@MN, with no observable needle damage under applied forces up to 1 N per needle (Figure 4C). At a displacement depth of 400 µm, the measured fracture forces for HA/MN and Lu‐GAMP@MN were 0.39 and 0.48 N/needle, respectively, both significantly higher than the minimum threshold of 0.045 N/needle required for effective skin penetration [48]. We next evaluated the cumulative release profile of ^177^Lu‐GAMP in vitro. Upon immersion of the microneedles in PBS, ^177^Lu‐GAMP was released rapidly from the microneedle matrix within the first 20 min, reaching approximately 60%, followed by a more gradual release that approached 80% by 120 min (Figure S20), suggesting that ^177^Lu‐GAMP was released in a sustained manner as the HA matrix gradually dissolves.

The ability of Lu‐GAMP@MN to penetrate the skin was further explored in mice. H&E staining showed distinct microneedle insertion profiles with an average penetration depth of 229.2 µm (Figure 4D). To assess dissolution kinetics, Lu‐GAMP@MN was applied to mouse skin for varying durations. Optical microscopy revealed that the needle tips began dissolving within 10 min and were completely dissolved after 60 min (Figure 4E). To evaluate biodistribution, NR‐labeled Lu‐GAMP was loaded into microneedles and applied to B16‐F10 subcutaneous melanoma‐bearing mice. Fluorescence microscopy confirmed successful encapsulation of NR‐labeled Lu‐GAMP, with strong red fluorescence observed in the microneedles upon excitation (Figure 4F). In vivo fluorescence imaging demonstrated sustained localization of NR‐labeled Lu‐GAMP at the tumor site for over 96 h after application (Figure 4G and Figure S21), which may be attributed to the multichannel delivery nature of microneedles, allowing broader drug dispersion within the lesion. Gamma counter analysis was performed to measure ^177^Lu activity in major organs and tumor tissue. Results showed high radioactivity localized at the tumor site after applying the microneedle with minimal accumulation in the heart, liver, spleen, lungs, and kidneys during the 192‐h monitoring period (Figure 4H and Table S4). These findings indicate prolonged tumor retention of ^177^Lu‐GAMP@MN and suggest the potential for improved anticancer efficacy with reduced systemic toxicity.

### 2.5 ^177^Lu‐GAMP@MN Suppresses Subcutaneous Melanoma Growth In Vivo

**FIGURE 5:**
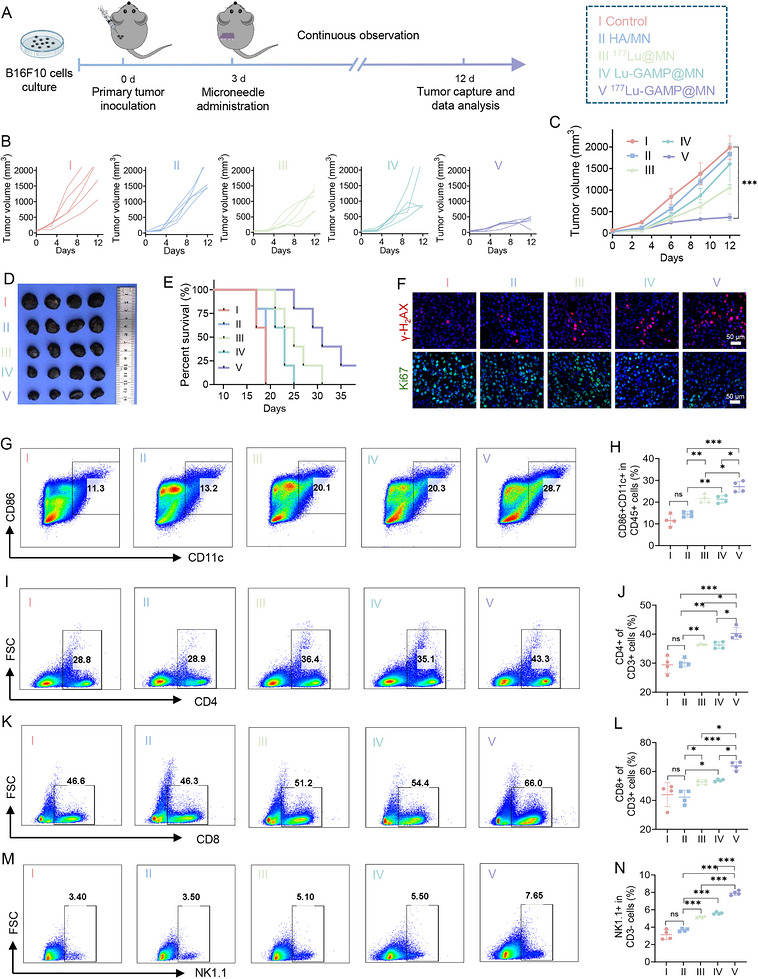
(A) Schematic illustration of ^177^Lu‐GAMP@MN‐mediated antitumor therapy in B16‐F10 tumor‐bearing mice. (B) Individual and (C) average tumor growth curves across different treatment groups (*n* = 5). (D) Representative images of excised tumors after 12 days of treatment (*n* = 4). (E) Kaplan–Meier survival curves of mice receiving various therapies (*n* = 5). (F) Immunofluorescence staining of γ‐H_2_AX and Ki67 in tumor tissues harvested after treatment. (G) Representative flow cytometry scatter plots and (H) quantification of CD11c^+^CD86^+^ dendritic cells within tumor tissues (*n* = 4). (I) Representative scatter plots and (J) corresponding quantification of CD3^+^CD4^+^ helper T cells within tumor tissues (*n* = 4). (K) Representative scatter plots and (L) quantification of CD3^+^CD8^+^ cytotoxic T cells within tumor tissues (*n* = 4). (M) Representative scatter plots and (N) quantification of NK cell populations within tumor tissues (*n* = 4). Data are presented as the mean ± SD. ns, not significant (*p* > 0.05), *p < 0.05, **p < 0.01, ***p < 0.001.

To evaluate the in vivo antitumor efficacy of ^177^Lu‐GAMP@MN, a subcutaneous melanoma model was established by inoculating B16‐F10 cells into C57BL/6 mice (Figure 5A). Once tumors reached approximately 50 mm^3^ in volume, the mice were randomly divided into five groups: 1) control group, the mice without any treatment; 2) HA/MN, the blank microneedle; 3) ^177^Lu@MN, the ^177^Lu‐loaded microneedle; 4) Lu‐GAMP@MN, the Lu‐GAMP‐loaded microneedle; 5) ^177^Lu‐GAMP@MN, the ^177^Lu‐GAMP‐loaded microneedle. Tumor growth was monitored over a 12‐day period. The HA/MN group showed tumor progression comparable to the control group, indicating negligible therapeutic benefit (Figure 5B,C). In contrast, treatment with ^177^Lu‐GAMP@MN resulted in pronounced tumor suppression, with the tumor growth inhibition (TGI) value of 77.8% following a single administration (Figure S22). Moderate inhibitory effects were observed in the ^177^Lu@MN and Lu‐GAMP@MN groups. Representative tumor images further confirmed the superior efficacy of ^177^Lu‐GAMP@MN (Figure 5D). Moreover, mice treated with ^177^Lu‐GAMP@MN demonstrated significantly prolonged survival compared to other groups (Figure 5E and Table S5). Immunofluorescence analysis of tumor sections revealed increased γ‐H_2_AX expression and reduced Ki67 levels in the ^177^Lu‐GAMP@MN group, indicating extensive DNA damage and suppressed tumor cell proliferation (Figure 5F and Figure S23). Serum biomarkers (AST, ALT, BUN, and CREA) remained within normal ranges, and body weight remained stable throughout treatment (Figures S24 and S25), suggesting excellent biocompatibility of ^177^Lu‐GAMP@MN.

In addition, the activation of the STING pathway in vivo was confirmed by the upregulation of p‐STING, p‐TBK1, p‐IRF3, and IFN‐β (Figure S26). Western blot analysis revealed that the phosphorylation levels of STING and its downstream signaling molecules, TBK1 and IRF3 in tumor tissues in the ^177^Lu‐GAMP@MN group were significantly increased, indicating effective signal transduction. Meanwhile, the elevated expression of IFN‐β, a key downstream effector, further verified the functional activation of the STING pathway and its role in initiating innate immune responses. Next, the infiltration of immune cells into tumor sites after treatment was analyzed via flow cytometry 2 days post‐treatment. Mice treated with ^177^Lu‐GAMP@MN showed a significant increase in CD11c^+^CD86^+^ mature DCs (Figure 5G,H), indicating enhanced antigen presentation. Simultaneously, this treatment promoted the infiltration of CD3^+^CD4^+^ helper T cells and CD3^+^CD8^+^ cytotoxic T cells (Figure 5I–L). A notable reduction in immunosuppressive myeloid‐derived suppressor cells (MDSCs, CD11b^+^Gr‐1^+^) was observed in the ^177^Lu‐GAMP@MN group (Figure S27). The proportion of natural killer (NK) cells was also elevated (Figure 5M,N), reflecting enhanced innate immune activation. Together, these results demonstrate that ^177^Lu‐GAMP@MN not only suppresses melanoma growth but also elicits a robust antitumor immune response, contributing to its overall therapeutic efficacy.

### 2.6 In Vivo Synergistic Antitumor Efficacy of ^177^Lu‐GAMP@MN Combined With aPD‐L1 Immune Checkpoint Blockade

**FIGURE 6:**
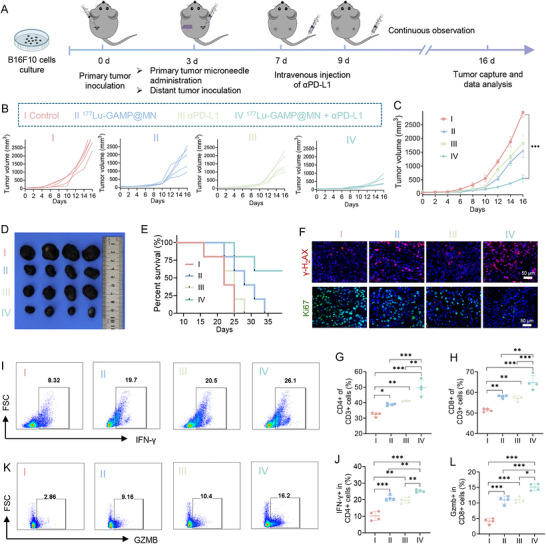
(A) Schematic diagram illustrating the treatment strategy combining ^177^Lu‐GAMP@MN and aPD‐L1 for the management of both primary and distant tumors. (B) Individual and (C) average tumor growth curves across different treatment groups (*n* = 5). (D) Representative images of excised tumors after 16 days of treatment (*n* = 4). (E) Kaplan–Meier survival curves of mice receiving different therapeutic regimens (*n* = 5). (F) Immunofluorescence staining of γ‐H_2_AX and Ki67 in tumor tissues collected post‐treatment. Quantification of tumor‐infiltrating (G) CD3^+^CD4^+^ helper T cells and (H) CD3^+^CD8^+^ cytotoxic T cells (*n* = 4). (I) Representative flow cytometry plots and (J) quantification of IFN‐γ^+^CD4^+^ T cells within tumor tissues (*n* = 4). (K) Representative flow cytometry plots and (L) quantification of GZMB^+^CD8^+^ T cells within tumor tissues (*n* = 4). Data are presented as the mean ± SD. *p < 0.05, **p < 0.01, ***p < 0.001.

Combining therapeutic modalities presents a compelling strategy to improve the efficacy of ICIs, especially those targeting PD‐L1, by engaging complementary immunomodulatory mechanisms. To evaluate this strategy, we conducted an in vivo study to examine the synergistic antitumor effects of ^177^Lu‐GAMP@MN in combination with aPD‐L1 therapy (Figure 6A). After 16 days of treatment, mice receiving the combination therapy exhibited significantly reduced primary tumor volumes, achieved a TGI of 82.1% in primary tumors, and showed delayed tumor progression compared to those receiving monotherapy (Figures 6B–D and Figure S28). Furthermore, the combined treatment significantly extended overall survival (Figure 6E and Table S6). Immunofluorescence staining revealed increased γ‐H_2_AX expression and reduced Ki67 levels in the combination group, indicating enhanced DNA damage and suppressed tumor cell proliferation, likely due to radiation‐induced genotoxic stress (Figure 6F and Figure S29). To elucidate the immune mechanisms driving these effects, we analyzed tumor‐infiltrating lymphocytes and effector cytokine expression. Flow cytometry revealed a notable increase in CD3^+^CD4^+^ helper T cells and CD3^+^CD8^+^ cytotoxic T cells, particularly in the combination group (Figure 6G,H). Moreover, ^177^Lu‐GAMP@MN combined with aPD‐L1 markedly elevated the levels of IFN‐γ^+^CD4^+^ T cells and GZMB^+^CD8^+^ T cells (Figure 6I–L), reflecting enhanced T cell activation and cytolytic function. Collectively, these findings demonstrate that the combined approach not only promotes T cell infiltration into the TME but also boosts their effector functions, thereby amplifying immune‐mediated tumor eradication.

### 2.7 Boosting Abscopal Effects by Combining ^177^Lu‐GAMP@MN With aPD‐L1

**FIGURE 7:**
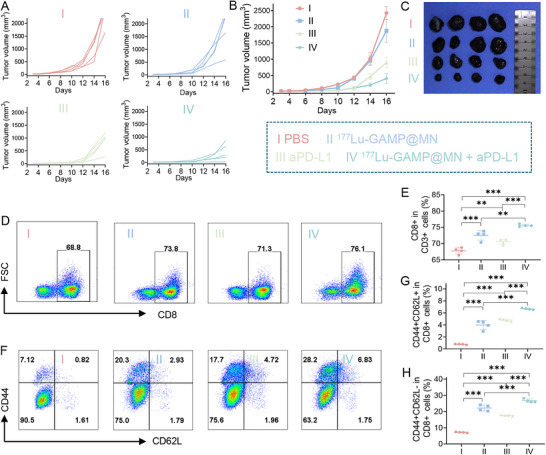
(A) Individual and (B) average growth curves of distant tumors following various treatments (*n* = 5). (C) Photographs of excised tumors post‐treatment (*n* = 4). (D) Representative flow cytometry scatter plots and (E) quantification of CD3^+^CD8^+^ cytotoxic T cells infiltrating distant tumor tissues (*n* = 4). (F) Representative scatter plots showing the distribution of central memory T cells (TCM, CD44^+^CD62L^+^) and effector memory T cells (TEM, CD44^+^CD62L^−^) in tumors harvested after treatment. Quantification of (G) TCM and (H) TEM populations in distant tumors (*n* = 4). Data are presented as the mean ± SD. *p < 0.05, **p < 0.01, ***p < 0.001.

Encouraged by the potent antitumor immune responses elicited by ^177^Lu‐GAMP@MN, we next investigated its ability to induce abscopal effects when combined with aPD‐L1. An artificial metastatic B16‐F10 tumor model was established by inoculating a secondary tumor on the contralateral flank following microneedle therapy of the primary tumor (Figure 6A). While monotherapy with either ^177^Lu‐GAMP@MN or aPD‐L1 partially suppressed distant tumor growth, the combination therapy produced a synergistic antitumor response, resulting in enhanced regression of both primary and distant tumors compared to either treatment alone (Figure 7A–C). Immune profiling further revealed substantial remodeling of the tumor immune microenvironment. Flow cytometry showed that both monotherapies modestly increased CD8^+^ cytotoxic T cell infiltration, whereas the combination treatment caused a marked CD8^+^ T cell accumulation (Figure 7D,E), suggesting synergistic activation and recruitment of cytotoxic lymphocytes. Further immunophenotyping highlighted critical changes in memory T cell subsets, including central memory T cells (TCM, CD44^+^CD62L^+^) and effector memory T cells (TEM, CD44^+^CD62L^−^), which contribute to long‐term immune surveillance and rapid recall responses, respectively. Although all treatment groups induced TCM and TEM infiltration, the combination therapy markedly expanded the total CD8^+^ memory T cell population, with a predominance of the TEM subset (Figure 7F–H). This TEM‐biased profile suggests that ^177^Lu‐GAMP@MN combined with aPD‐L1 not only improves tumor eradication but also reprograms systemic antitumor immunity to support long‐term immunological memory and reduce the risk of metastasis or recurrence.

## 3 Conclusion

In this study, we developed a novel radiolabeled CPs‐based microneedle platform (^177^Lu‐GAMP@MN) that integrates localized brachytherapy with immune modulation to improve melanoma treatment. The ^177^Lu‐GAMP complex, formed through the self‐assembly of ^177^Lu^3+^ with nucleotide ligands, effectively activates the STING pathway and induces GSDME‐mediated pyroptosis, thereby providing robust antitumor immune responses. Incorporation of ^177^Lu‐GAMP into dissolvable microneedles enables precise transdermal delivery with efficient retention at tumor site while ensuring safety. In vivo studies demonstrated that ^177^Lu‐GAMP@MN not only suppressed melanoma growth and improved overall survival but also facilitated DC maturation and enhanced T cell and NK cell infiltration. Furthermore, combination with aPD‐L1 therapy resulted in synergistic therapeutic effects, reinforced T cell functionality, and durable immunological memory, demonstrating significant inhibition of both primary and distant tumors in murine models. These findings highlight the potential of ^177^Lu‐GAMP@MN as a minimally invasive, multifunctional transdermal platform for enhancing melanoma immunotherapy.

## 4 Materials and Methods

### 4.1 Materials

Adenosine monophosphate (AMP) and Guanidine monophosphate (GMP) were purchased from MedChemExpress Co., Ltd (Shanghai, China). Polydimethylsiloxane (PDMS) was purchased from Chipscreen Co., Ltd (Taizhou, China). Hyaluronic acid (HA, Mw ∼10 kDa) was purchased from MeilunBio Co., Ltd (Dalian, China). ^177^Lu was purchased from HTA Co., Ltd (Beijing, China). DMEM, RPMI 1640, annexin V‐FITC apoptosis detection kit, and 2′,7′‐Dichlorofluorescin (DCFH‐DA) were supplied by Solarbio Co., Ltd (Beijing, China). Bovine serum albumin (BSA), methyl thiazolyl tetrazolium (MTT), 4′,6‐diamidino‐2‐phenylindole (DAPI) and Calcein AM/PI staining kit were obtained from Beyotime Co., Ltd (Shanghai, China). Nile red (NR) was obtained from Sinopharm Co., Ltd (Shanghai, China). All antibodies for Western blot were obtained from Cell Signaling Technologies (CST, US). All antibodies for flow cytometry were obtained from BioLegend Co., Ltd (US). aPD‐L1 was obtained from Bio X Cell Co., Ltd (US).

### 4.2 Preparation and Characterization of ^177^Lu‐GAMP

Lu‐GAMP was synthesized via a self‐assembly method. Specifically, 100 µL each of AMP and GMP solutions (100 mm) were added to 5 mL of ultrapure water and vigorously mixed. Subsequently, 200 µL of LuCl_3_ solution (100 mm) was slowly introduced under continuous stirring. After an additional 30 min of stirring, the mixture was centrifuged at 10000 rpm for 10 min, and the supernatant was discarded. The resulting precipitate was redispersed in 5 mL of water and dissolved under stirring. The solution was then subjected to ultrasonication in an ice bath to obtain Lu‐GAMP. For the NR‐labeled Lu‐GAMP preparation, 10 µL of NR solution (5 mg mL^−1^) was mixed with Lu‐GAMP and then incubated under gentle stirring at room temperature for 30 min. The mixture was centrifuged to collect the NR‐loaded nanoparticles, and the supernatant containing free NR and residual solvent was discarded. The nanoparticles were resuspended in fresh deionized water. This washing process was repeated several times to ensure complete removal of unloaded NR. Additionally, ^177^Lu‐GAMP was prepared by radiolabeling Lu‐GAMP with a trace amount of [^177^Lu]LuCl_3_ using the same self‐assembly synthesis protocol. Concretely, 100 µL each of AMP and GMP solutions (100 mm) were added to 5 mL of ultrapure water and vigorously mixed. Afterwards, 200 µL of [^177^Lu]LuCl_3_ (40 mCi mL^−1^, 100 mm) was slowly introduced under continuous stirring for 30 min. Then the ^177^Lu‐GAMP was isolated by centrifugation (12,000 rpm, 15 min), and resuspended it in 5 mL of water and sonicated to disperse it evenly.

The particle size and polydispersity index (PDI) of the Lu‐GAMP were measured using dynamic light scattering (DLS, Malvern, UK), while its morphology and surface elemental distribution were characterized by transmission electron microscopy coupled with energy‐dispersive X‐ray spectroscopy (TEM‐EDS, Tecnai G2 F20, FEI, US). The radiolabeling rate and radiolabeling stability of ^177^Lu‐GAMP was measured using gamma counter (WIZARD, PerkinElmer, US).

### 4.3 Cell Culture

The B16‐F10 mouse melanoma cells and DC 2.4 mouse dendritic cells were obtained from the American Type Culture Collection (ATCC). The B16‐F10 cells were cultured in DMEM medium supplemented with 10% FBS at 37°C in a humidified incubator with 5% CO_2_, and the DC 2.4 cells were cultured in 1640 medium supplemented with 10% FBS at 37°C in a humidified incubator with 5% CO_2_. Cells were regularly monitored and passaged to maintain optimal growth conditions.

### 4.4 Cellular Uptake and Lysosomal Escape

B16‐F10 cells were seeded into confocal culture dishes and incubated overnight. Then, cells were treated with NR‐labeled Lu‐GAMP for 0.5, 1, 2, 4, or 6 h. After washing with PBS, the cells were stained with LysoTracker and Hoechst 33342, then imaged using a confocal laser scanning microscope (CLSM, Zeiss, Germany).

### 4.5 Quantitative Real‐Time Polymerase Chain Reaction (qRT‐PCR)

Total RNA was extracted using RNAiso Plus reagent (Takara Bio Inc.), and complementary DNA (cDNA) was synthesized with the PrimeScript RT reagent kit (Takara Bio Inc.). Quantitative real‐time PCR was then performed using SYBR Green reagent (Bimake) in a two‐step protocol. Gene expression levels were analyzed using the 2^−^ *^ΔΔCt^* method and normalized to β‐actin as the internal control.

### 4.6 Western Blot

After extracting proteins from cell lysates, their concentrations were determined using the Bicinchoninic Acid (BCA) Assay Kit (Beyotime). Proteins were separated by electrophoresis on a 12% SDS‐PAGE gel, transferred onto a polyvinylidene difluoride (PVDF) membrane, and blocked with 5% skim milk. The membrane was then incubated with primary antibodies for STING, p‐STING, GSDME, N‐GSDME, TBK1, p‐TBK1, GAPDH, and β‐actin overnight at 4°C, followed by incubation with appropriate secondary antibodies. Protein bands were visualized using an enhanced chemiluminescence (ECL) imaging system.

### 4.7 Molecular Docking Study

Molecular docking analyses were performed using Discovery Studio 3.1. The crystal structures of murine STING (PDB ID: 4KC0) and human STING (PDB ID: 4EMU) were retrieved from the Protein Data Bank. The Lu‐GAMP ligand and receptor proteins were prepared and energy‐minimized within Discovery Studio using the CHARMm force field. Docking simulations were executed using the GOLD program. The binding interactions and conformations were subsequently visualized using PyMOL.

### 4.8 Cell Viability Assay

B16‐F10 cells were seeded in 96‐well plates at a density of 2000 cells per well and incubated overnight to allow cell attachment. The next day, cells were treated with various formulations (^177^Lu, Lu‐GAMP, ^177^Lu‐GAMP) for 48 h. Following treatment, the culture medium was removed, and 100 µL of MTT solution (5 mg mL^−1^) was added to each well. After a 4‐h incubation at 37°C, the absorbance at 570 nm was measured using a microplate reader (EPOCH‐SN, BioTek, USA).

### 4.9 Detection of LDH Release

LDH levels in the culture supernatants were measured using the LDH Cytotoxicity Assay Kit (Promega, USA) in accordance with the manufacturer's instructions. Following 24‐h treatment with varying concentrations of ^177^Lu, 120 µL of supernatant was transferred to 96‐well plates. Then, 60 µL of LDH detection reagent was added to each well and incubated for 30 min. Absorbance was subsequently measured at 490 nm using a microplate reader.

### 4.10 Live/Dead Cell Staining Assay

B16‐F10 cells were seeded onto cell culture slides placed in a 12‐well plate. Once the cells reached approximately 70% confluency, 1 µL of Calcein‐AM was added, mixed thoroughly by pipetting, and incubated at 37°C in the dark for 25 min. Subsequently, 3 µL of propidium iodide (PI) was added to the stained cells and incubated at room temperature in the dark for an additional 5 min. Following staining, the cells were centrifuged to remove the staining solution, and the slides were transferred onto glass microscope slides. Coverslips were gently applied, and the samples were observed under an inverted fluorescence microscope (Leica, Germany) to identify live cells (green fluorescence) and dead cells (red fluorescence).

### 4.11 PI‐Positive Flow Cytometry Analysis

B16‐F10 cells were seeded into six‐well plates and treated with different formulations for 24 h. After incubation, the cells were collected in centrifuge tubes. They were then stained with propidium iodide (PI) in the dark for 5 min. The stained samples were analyzed by flow cytometry using a BD Biosciences system (USA).

### 4.12 Intracellular ROS Detection

B16‐F10 cells were seeded in six‐well plates and incubated overnight. The following day, cells were treated with various formulations and further incubated for 24 h. After treatment, cells were washed and incubated with DCFH‐DA (10 µm) in PBS for 20 min in the dark. Fluorescence signals were subsequently observed using an inverted fluorescence microscope.

### 4.13 Immunofluorescence Staining

Glass coverslips were pre‐coated with poly‐l‐lysine (Procell, China) and placed in six‐well plates. B16‐F10 cells were seeded onto the coated slides and incubated overnight. Then, the cells were treated with various formulations for 24 h After treatment, the slides were washed and fixed with 4% paraformaldehyde. Permeabilization was achieved using 0.2% Triton X‐100 for 20 min, followed by blocking with 10% goat serum for 30 min. The slides were then incubated overnight at 4°C with diluted primary antibodies targeting γ‐H_2_AX. Subsequently, a secondary antibody was added and incubated at 37°C for 1 h. Finally, the nuclei were counterstained with DAPI, and fluorescence signals were captured using a confocal laser scanning microscope (CLSM, ZEISS, Germany).

### 4.14 DCs Maturation

DC 2.4 cells were seeded into six‐well plates and treated with various formulations for 24 h. After incubation, DC 2.4 cells were collected, washed with PBS, and resuspended in fresh PBS. To block Fc receptors, the cell suspension was incubated with CD16/32, followed by staining with a panel of fluorophore‐labeled antibodies (anti‐Gr1‐BV510, anti‐MHC II‐FITC, anti‐CD11c‐APC, and anti‐CD86‐PE‐CY7) in the dark for 30 min. Flow cytometry was then conducted using a BD Biosciences analyzer (USA).

### 4.15 Preparation and Characterization of ^177^Lu‐GAMP@MN

The ^177^Lu‐GAMP‐loaded microneedle (^177^Lu‐GAMP@MN) was prepared using a micro‐molding technique. Briefly, 1 g of hyaluronic acid (HA) powder was dissolved in 10 mL of PBS to obtain a 10% HA solution. ^177^Lu‐GAMP was added and uniformly dispersed by ultrasonication. A total of 100 µL of this matrix solution was poured into microneedle molds, ensuring complete filling of the cavities. The molds were centrifuged at 4500 rpm for 30 min and then vacuumed at room temperature for 10 min. After removing excess solution, a 50% HA solution was added, followed by another round of centrifugation and degassing. The molds were dried at 30°C for 12 h. The dried microneedles were then demolded to obtain the ^177^Lu‐GAMP@MN.

The radiolabeling efficiency of ^177^Lu‐GAMP within the microneedles was determined using a gamma counter. Morphological features and dimensional analysis were conducted via scanning electron microscopy (SEM). To examine their transdermal penetration and degradation behavior, the microneedles were applied to the dorsal skin of mice. At designated time points (0, 5, 10, 20, 40, and 60 min), microneedles were removed and their structure assessed using an inverted optical microscope. Skin samples were fixed in paraformaldehyde, stained with H&E, and evaluated microscopically for tissue integrity.

### 4.16 Animal Model

All animal procedures were approved by the Ethics Committee of Xiangya Hospital, Central South University, and conducted in strict accordance with the “3R” principle for animal experimentation (Ethics Code: 202411194). B16‐F10 cells were harvested, rinsed three times with PBS, and resuspended in cold serum‐free DMEM medium. A total of 1 × 10^6^ B16‐F10 melanoma cells in 100 µL of medium were subcutaneously injected into the right flank of 8‐week‐old female C57BL/6 mice (Shanghai SLAC Laboratory Animal Co., Ltd.). Once tumors became palpable, the mice were randomly assigned to treatment groups. Tumor size was measured every other day using a digital caliper, and volume was calculated as *V* = (length × width^2^)/2.

### 4.17 In Vivo Penetration Capability

After anesthetizing the mice, the dorsal skin was shaved and disinfected. The ^177^Lu‐GAMP@MN was applied to the treated area and removed at specific time points (5, 10, 30, and 60 min). Microneedle degradation was evaluated and recorded using an optical microscope (Scope.A1, ZEISS, Germany). To assess skin recovery, an additional microneedle patch was inserted into the dorsal skin and removed after 2 min. The excised skin tissue was fixed in 4% paraformaldehyde and subjected to H&E staining.

### 4.18 In Vivo Accumulation and Biodistribution Behavior

When tumor volumes reached approximately 50 mm^3^, NR‐labeled microneedle patches were applied to the tumor site and removed after 2 h. The intratumoral accumulation of ^177^Lu‐GAMP@MN was subsequently monitored using in vivo fluorescence imaging (S12‐FMT400010, PerkinElmer, USA). For biodistribution analysis, ^177^Lu‐GAMP@MN was administered via microneedle insertion into tumor‐bearing mice. At predetermined time points (2, 24, 48, 72, and 96 h), the mice were euthanized, and radioactivity levels in the heart, liver, spleen, lungs, kidneys, and tumor were quantified using a gamma counter.

### 4.19 In Vivo Antitumor Efficiency

When the tumor volume reaches approximately 50 mm^3^, tumor‐bearing mice are randomly allocated into five groups (*n* = 5): 1) control group, 2) HA/MN, 3) ^177^Lu@MN, 4) Lu‐GAMP@MN, 5) ^177^Lu‐GAMP@MN. Tumor dimensions and body weight are monitored every 3 days. On day 12 post‐treatment, mice are euthanized, and blood is collected via retro‐orbital bleeding, followed by centrifugation to obtain serum for biochemical analysis. Simultaneously, tumor tissues are harvested for flow cytometry. In survival studies, the experimental endpoint is defined by either spontaneous death or tumor progression exceeding 2000 mm^3^.

Furthermore, the in vivo antitumor efficacy of ^177^Lu‐GAMP@MN in combination with aPD‐L1 was evaluated against both primary and distant melanoma tumors. B16‐F10 cells suspended in PBS were subcutaneously injected into the upper left flank of mice to establish primary tumors, while additional cells were inoculated on the opposite flank to model distant tumors. Mice were randomly divided into four groups: 1) control, 2) ^177^Lu‐GAMP@MN alone, 3) aPD‐L1 alone, and 4) combined ^177^Lu‐GAMP@MN and aPD‐L1 (2.5 mCi kg^−1^). When the primary tumor reached approximately 50 mm^3^, the microneedle patch was applied, and distant tumor inoculation was performed on the same day. aPD‐L1 was administered intravenously on days 7 and 9. Tumor volume and body weight were recorded every 3 days, and mice were sacrificed on day 16 post‐treatment.

### 4.20 In Vivo Immunofluorescence Analysis

Excised tumor tissues were embedded in paraffin and sectioned. The sections were incubated at 60°C to facilitate deparaffinization, followed by blocking with 10% goat serum. Subsequently, the sections were incubated overnight at 4°C with primary antibodies targeting Ki67 and γ‐H_2_AX. After washing, appropriate secondary antibodies were applied. Nuclei were counterstained with DAPI, and fluorescence images were acquired using an inverted fluorescence microscope. Apoptotic cells were identified using a Colorimetric TUNEL Apoptosis Detection Kit (Beyotime, China) in accordance with the manufacturer's protocol.

### 4.21 In Vivo Flow Cytometry Analysis

Tumor tissues were enzymatically digested in DMEM and filtered through a cell strainer to generate single‐cell suspensions. The cells were subsequently treated with red blood cell lysis buffer. To assess DC maturation, CD4^+^ and CD8^+^ T cell, NK cell, MDSC, and memory T cell infiltration, and the activation of central and effector memory T cells, the suspensions were stained with corresponding antibodies. Flow cytometry was then conducted to analyze the immune cell populations.

### 4.22 Statistical Analyses

All data were analyzed using GraphPad Prism 8.0 and Image J 1.8.0 software. The measurement data, obtained from three or more independent repeated experiments, were expressed as mean ± standard deviation (mean ± SD). Paired Student's *t*‐test or one‐way analysis of variance (ANOVA) was employed for data analysis, with *p* < 0.05 considered statistically significant. The significance levels were denoted as follows: *p* < 0.05 (*), *p* < 0.01 (**), and *p* < 0.001 (***).

## Author Contributions

**Pian Yu**: methodology, experiment, funding acquisition, and writing. **Shijun Xiang**: data curation, experiment, and writing. **Lu Hao**: experiment, validation, and writing. **Jessica C. Hsu**: visualization and writing. **Kaixuan Li**: validation. **Rongxuan Yan**: experiment. **Ming Zhou**: conceptualization. **Yongxiang Tang**: visualization. **Ying Peng**: analytics. **Weibo Cai**: conceptualization. **Cong Peng**: methodology, review, and funding acquisition. **Peng Liu**: data analysis, visualization, writing, and funding acquisition. **Shuo Hu**: funding acquisition, resources, conceptualization, and supervision.

## Ethics Statement

All animal procedures were approved by the Ethics Committee of Xiangya Hospital, Central South University, and conducted in strict accordance with the “3R” principle for animal experimentation (Ethics Code: 202411194).

## Conflicts of Interest

Weibo Cai declares conflict of interest with the following corporations: Portrai, Inc., rTR Technovation Corporation, and Four Health Global Pharmaceuticals Inc. Weibo Cai is a member of the *Exploration* editorial board, and he was not involved in the handling or peer review process of this manuscript. All other authors declare no conflicts of interest.

## Supporting information




**Supporting File**: exp270170‐sup‐0001‐SuppMat.docx.

## Data Availability

The data that support the findings of this study are available from the corresponding author upon reasonable request.
