# Supplementary material for: Radiolabeled Coordination Polymer‐Loaded Microneedles for Synergistic Melanoma Brachytherapy–Immunotherapy via STING Activation and Pyroptosis
Source: Exploration (Beijing). 2026 Jun 22;6(3):20250737. doi: 10.1002/EXP.20250737 (PMC13317568; doi:10.1002/EXP.20250737)

**Supporting information**

**Radiolabeled Coordination Polymer-Incorporated Microneedle for Synergistic Melanoma Brachytherapy-Immunotherapy via STING Activation and Pyroptosis**


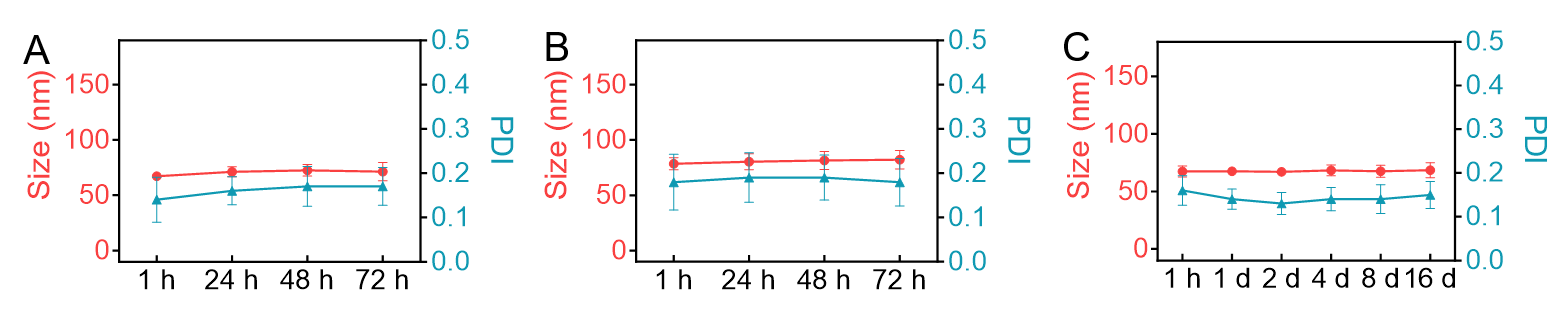


**Figure S1.** Temporal variations in the hydrodynamic diameter and PDI of Lu-GAMP incubated in (A) PBS supplemented with 10% FBS, (B) DMEM containing 10% FBS and (C) deionized water over a period of 16 days.


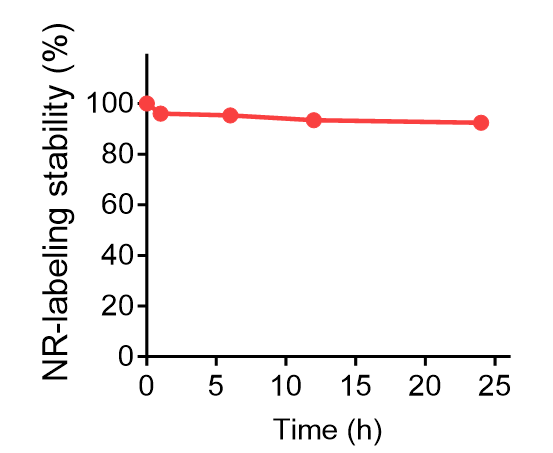


**Figure S2.** NR-labeling stability of NR-labeled Lu-GAMP in different time points.


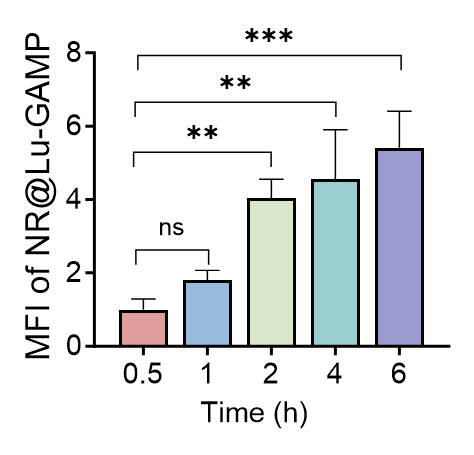


**Figure S3.** Quantitative analysis of the cellular uptake of NR-Labeled Lu-GAMP in B16-F10 cells.


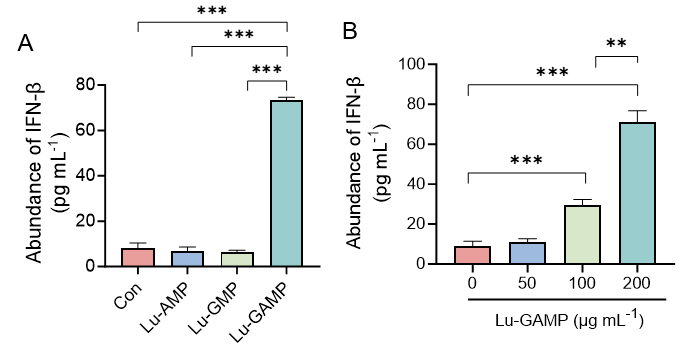


**Figure S4.** (A) IFN-β secretion from B16-F10 cells treated with Lu-AMP, Lu-GMP, and Lu-GAMP as measured by ELISA. (B) IFN-β secretion from B16-F10 cells treated with increasing concentrations of Lu-GAMP.


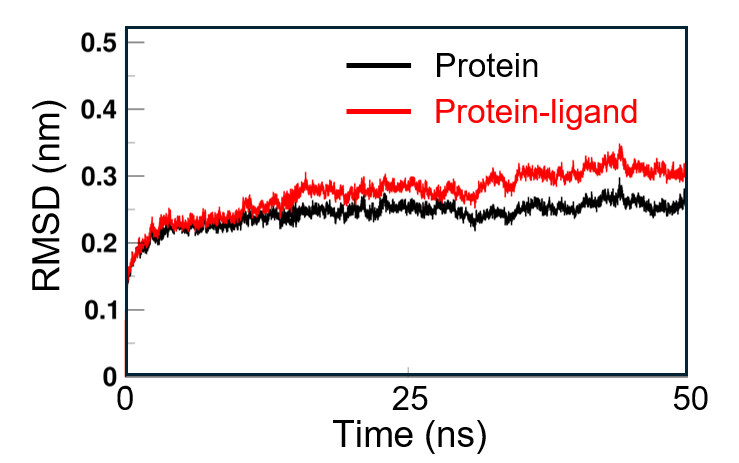


**Figure S5.** RMSD from molecular docking studies.


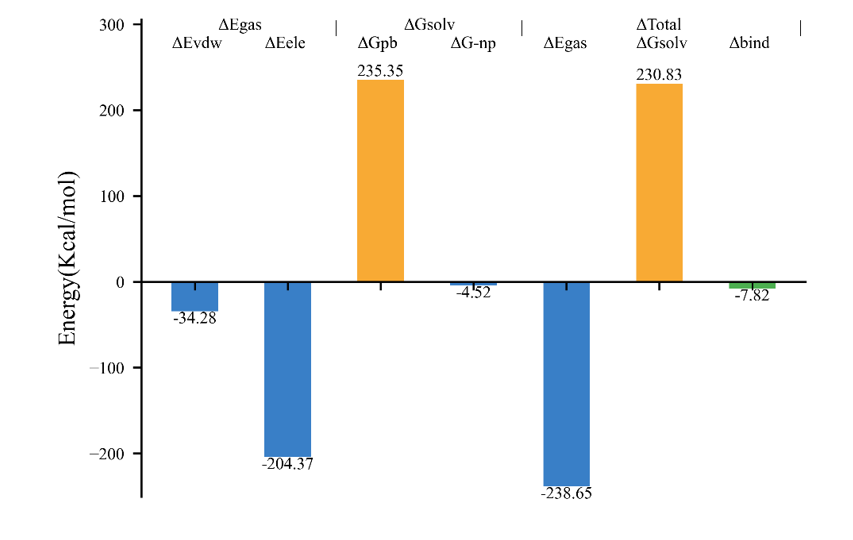


**Figure S6.** Binding energy scores from molecular docking studies.


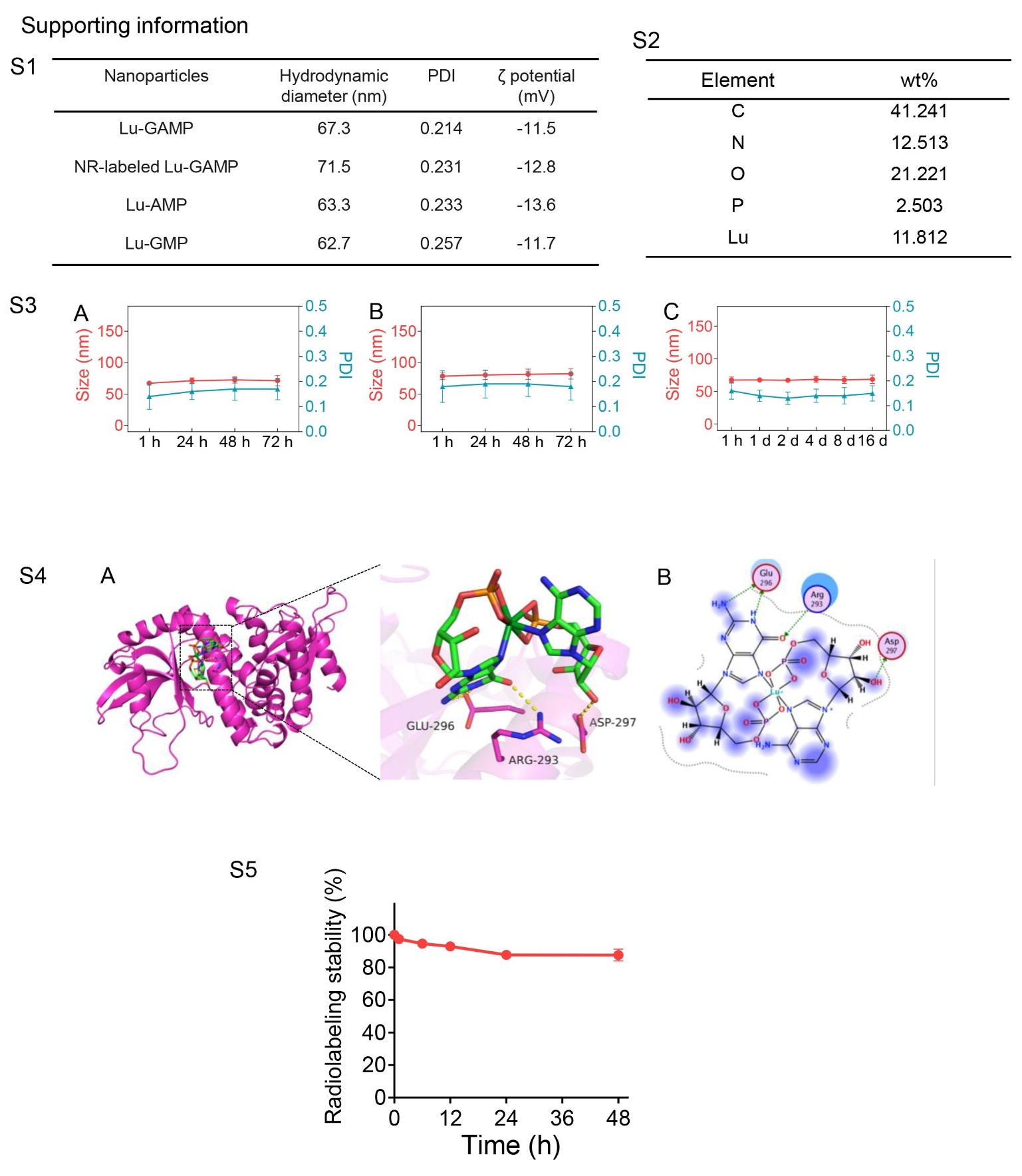


**Figure S7.** (A) The 3D binding mode and (B) 2D binding mode of Lu-GAMP in the binding site of hSTING.


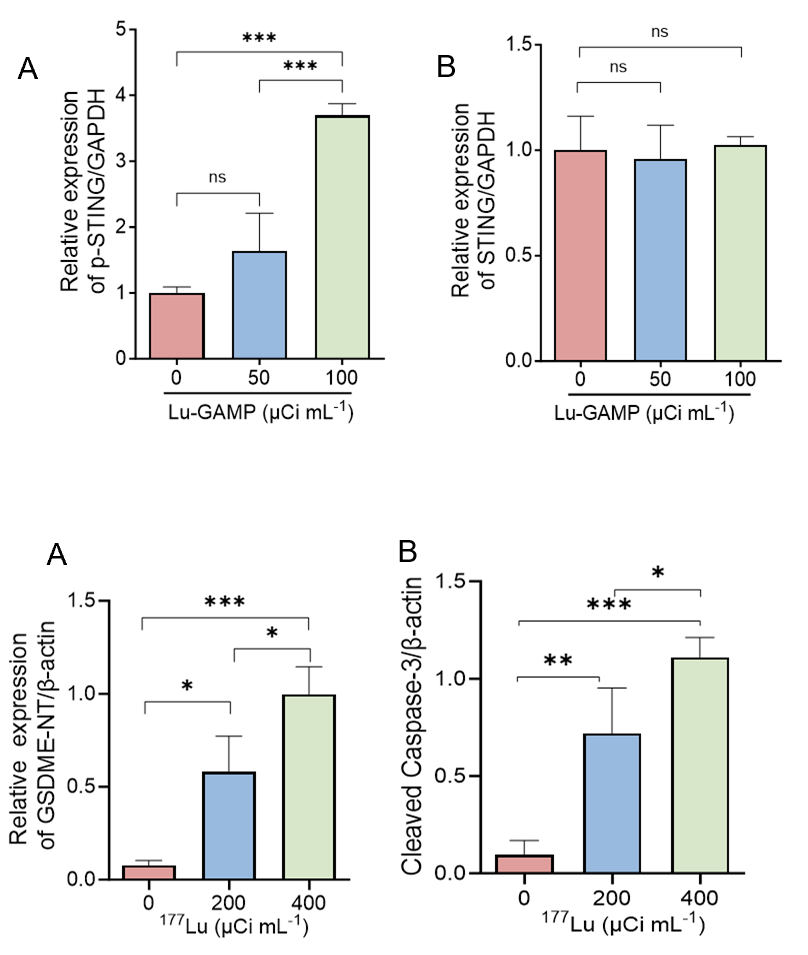


**Figure S8.** Quantification of (A) p-STING and (B) STING protein expression in B16-F10 cells following treatment with different concentrations of Lu-GAMP.


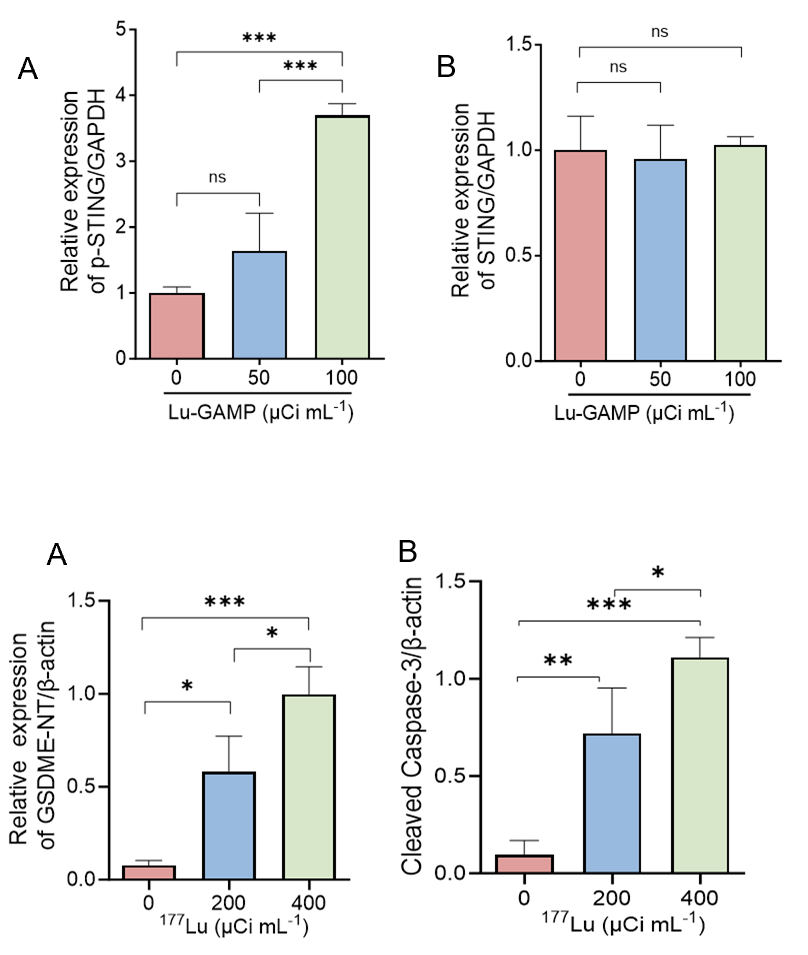


**Figure S9.** Quantification of (A) N-GSDME and (B) Cleaved Caspase-3 protein expression in B16-F10 cells following treatment with different concentrations of ^177^Lu.


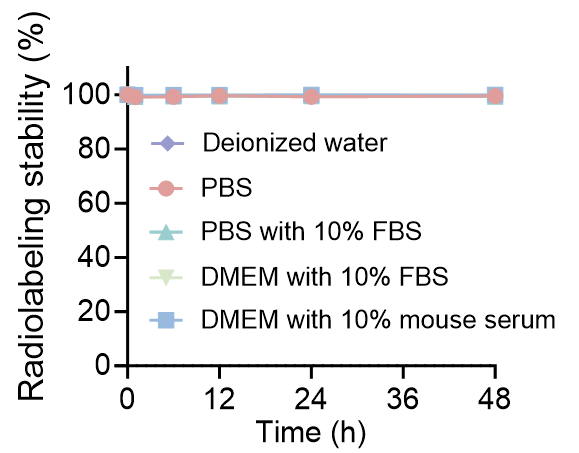


**Figure S10.** (A) The radiolabeling stability of ^177^Lu-GAMP in Deionized water, PBS, PBS with 10% FBS, DMEM with 10% PBS, and DMEM with 10% mouse serum.


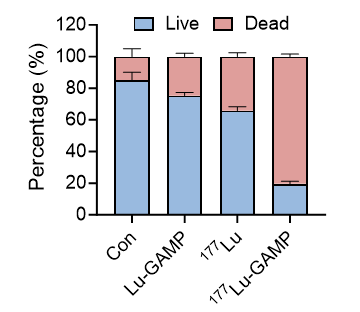


**Figure S11.** Quantitative analysis of live/dead cells following various treatments.


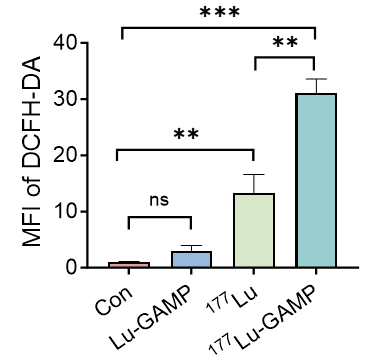


**Figure S12.** Quantitative analysis of B16-F10 cells stained with DCFH-DA after various treatments.


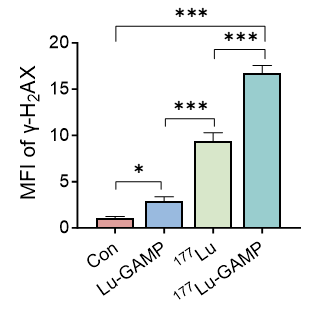


**Figure S13.** Quantitative analysis of B16-F10 cells stained with γ-H_2_AX after different treatments.


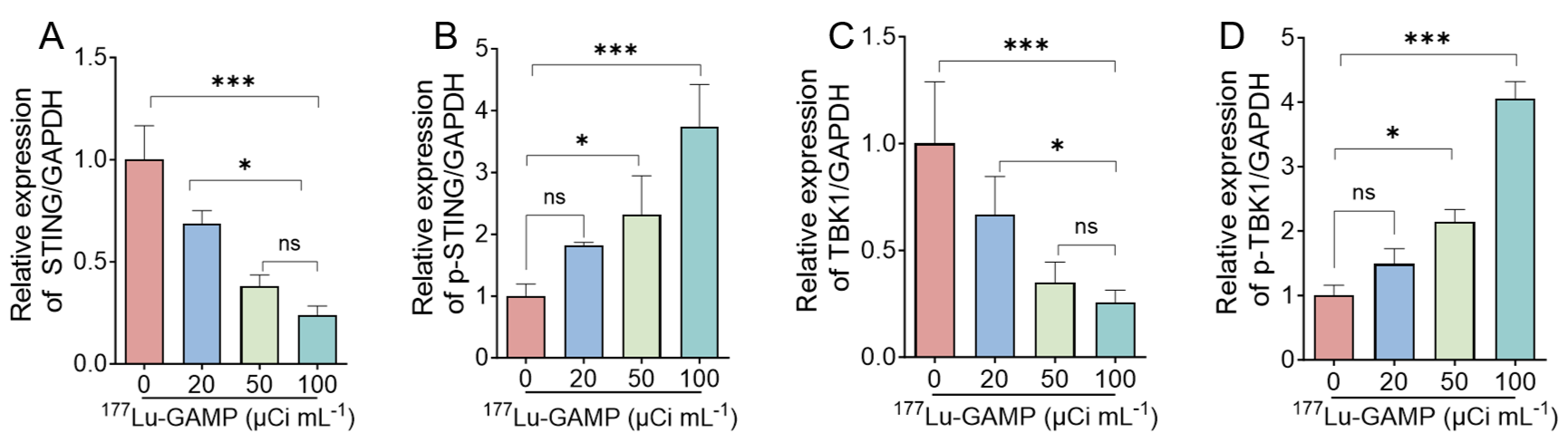


**Figure S14.** Quantification of (A) STING, (B) p-STING, (C) TBK1 and (D) p-TBK1 protein levels in B16-F10 cells following treatment with different concentrations of ^177^Lu-GAMP.


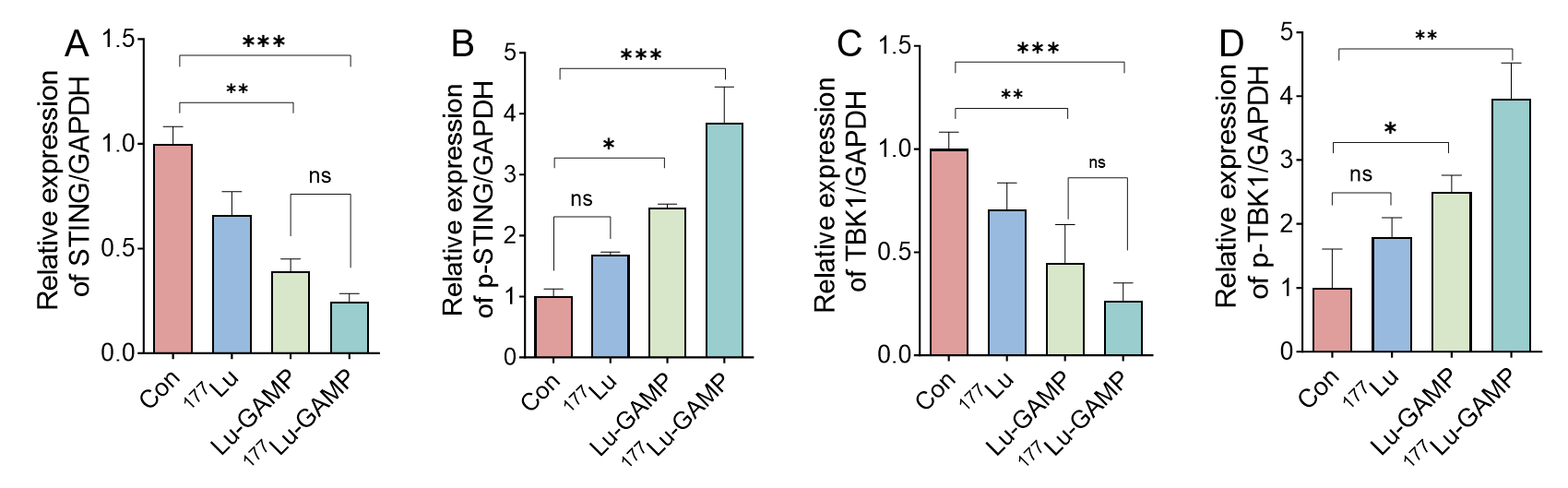


**Figure S15.** Quantification of (A) STING, (B) p-STING, (C) TBK1 and (D) p-TBK1 protein levels in B16-F10 cells following various treatments.


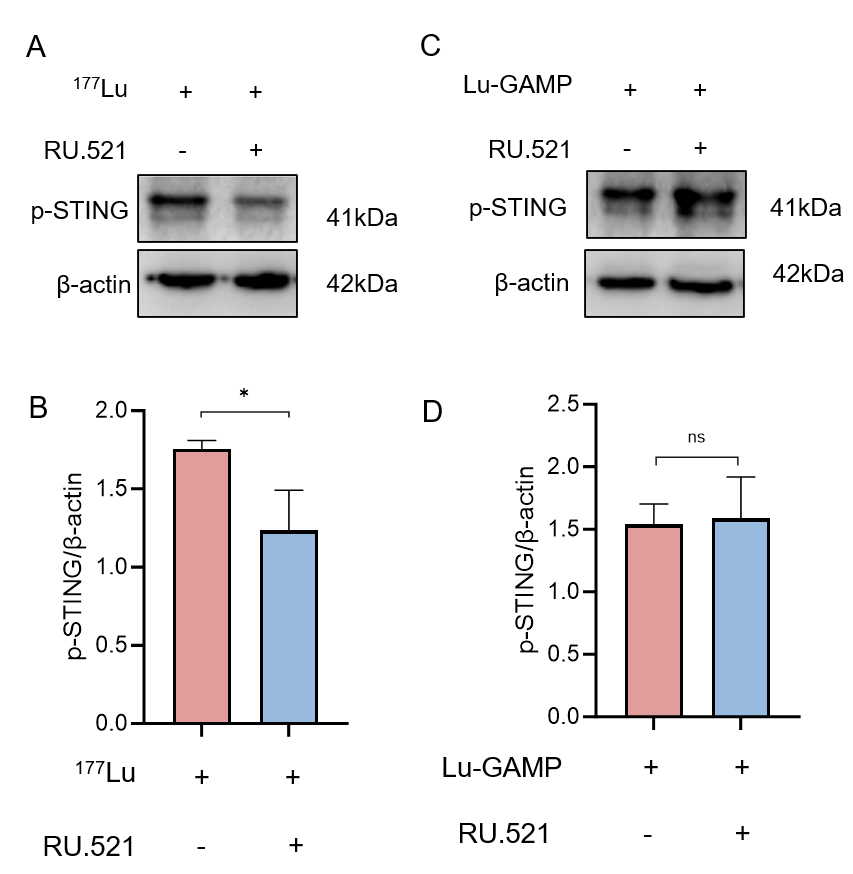


**Figure S16.** Western blot images of (A) p-STING and (B) quantification analysis treated with ^177^Lu alone or in combination with RU.521. Western blot images of (C) p-STING and (D) quantification analysis treated with Lu-GAMP alone or in combination with RU.521.


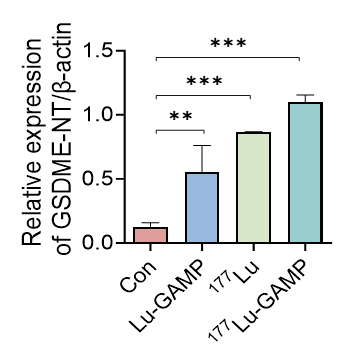


**Figure S17.** Quantification of N-GSDME in B16-F10 cells after different treatments.


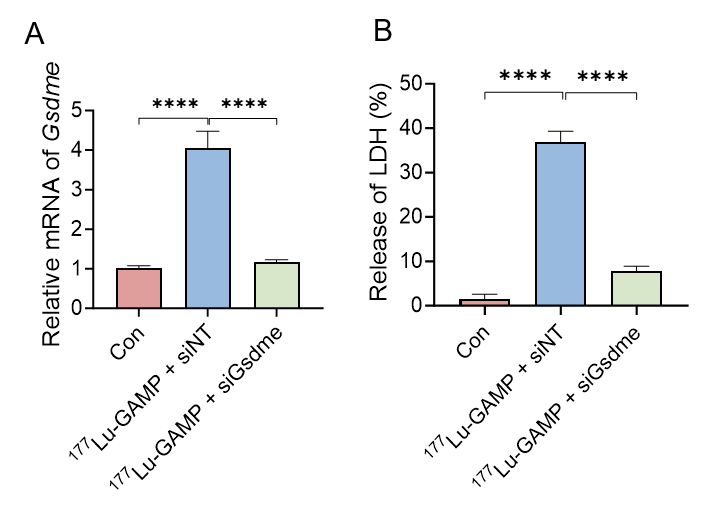


**Figure S18.** (A) RT-qPCR detection of *Gsdme* expression and (B) LDH release in the supernatant after treatment with siGsdme.


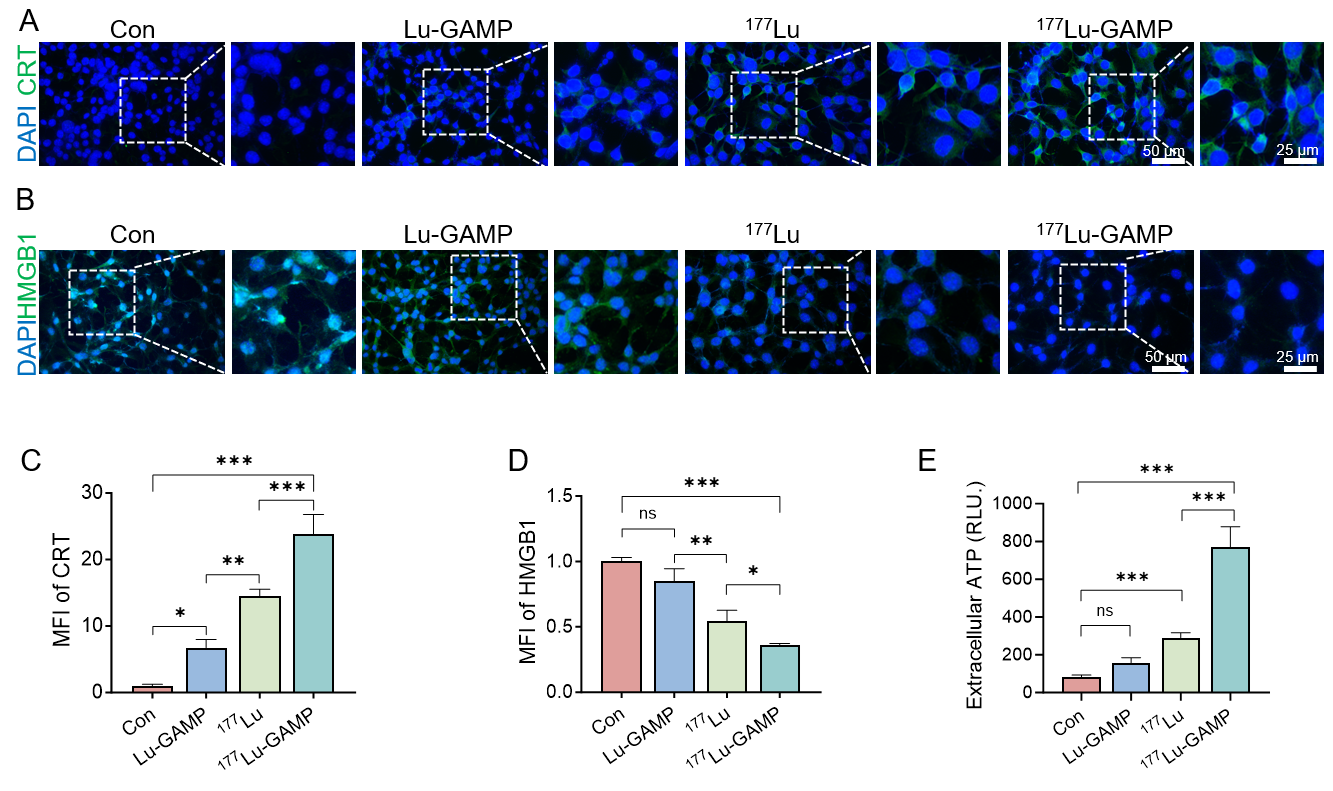


**Figure S19.** Fluorescence images of (A) CRT and (B) HMGB1 expression in B16-F10 cells after various treatments for 24 h. Fluorescence quantitative analysis of (C) CRT and (D) HMGB1 with various formulations. (E) ATP secretion in the B16-F10 cells supernatant after various treatments.


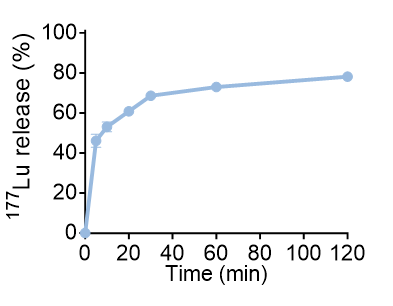


**Figure S20.** The cumulative release profile of ^177^Lu-GAMP in various time points.


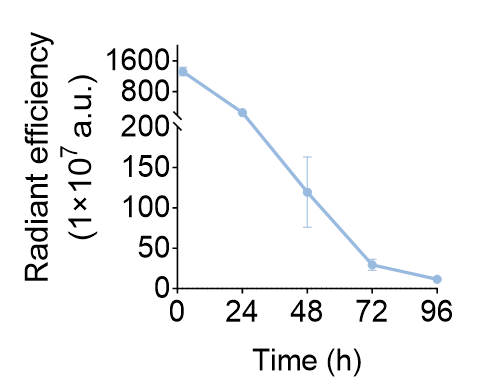


**Figure S21.** Quantitative analysis of fluorescence signals from NR-labeled Lu-GAMP@MN in subcutaneous B16-F10 tumor-bearing mice.


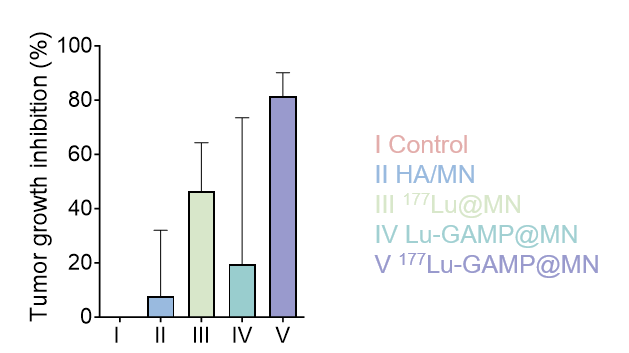


**Figure S22.** Tumor growth inhibition rate following different treatments.


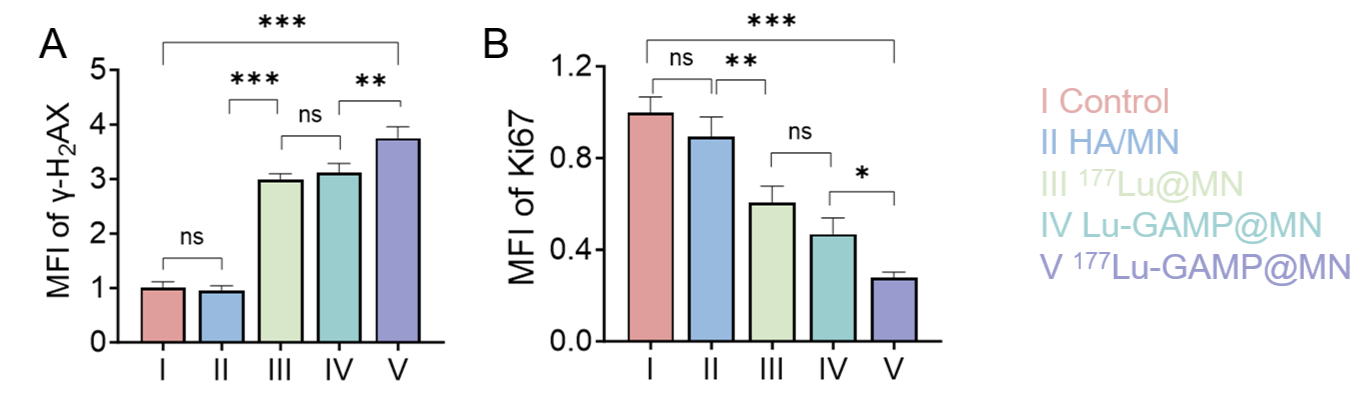


**Figure S23.** Quantitative analysis of (A) γ-H_2_AX and (B) Ki67 following various treatments.


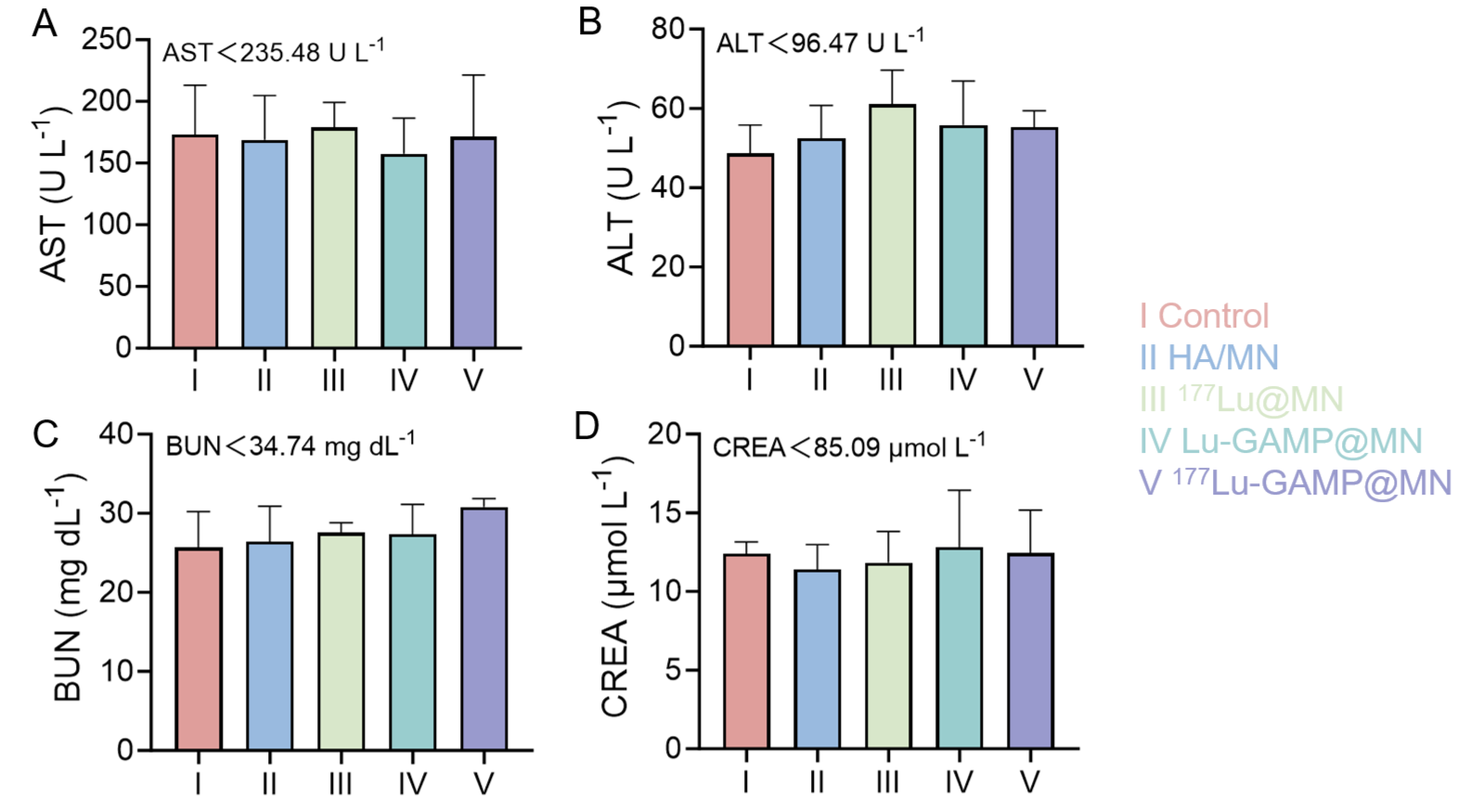


**Figure S24.** The serum levels of (A) AST, (B) ALT, (C) BUN and (D) CRE of mice after various treatments.


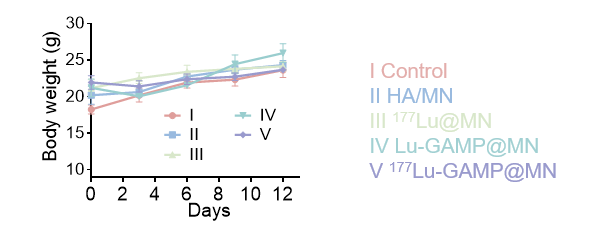


**Figure S25.** Average body weights of mice during various treatments.


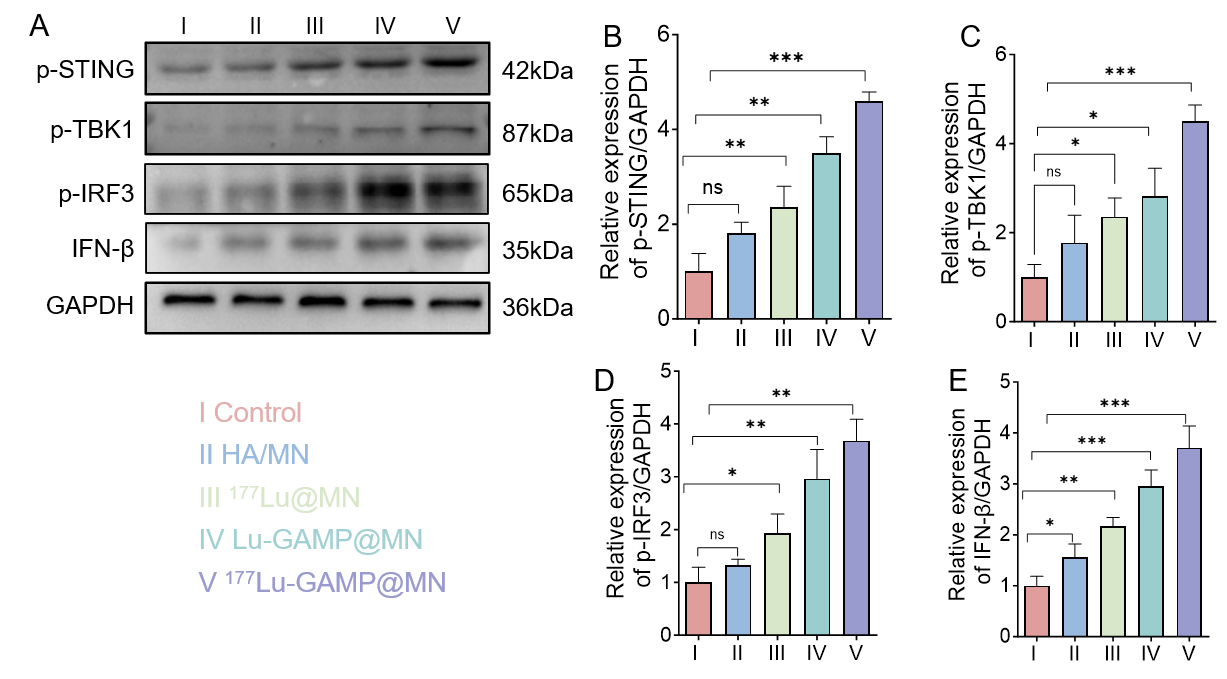


**Figure S26.** (A) Western blot images and quantification analysis of (B) p-STING, (C) p-TBK1, (D) p-IRF3 and (E) IFN-β following various treatments.


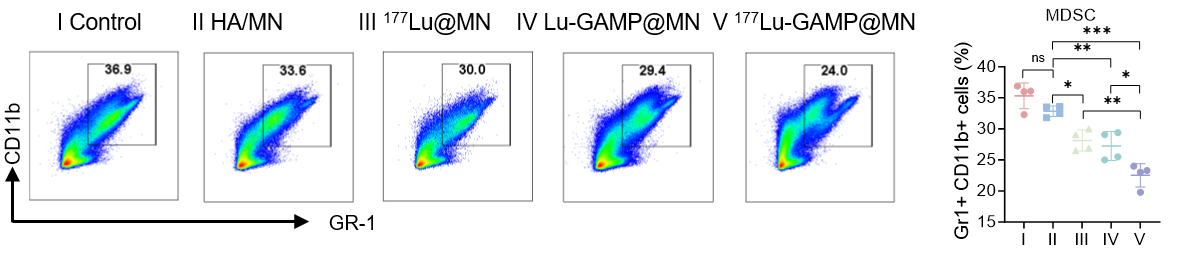


**Figure S27.** Representative scatter plots and quantification of GR-1^+^CD11b^+^ MDSC within tumor tissues.


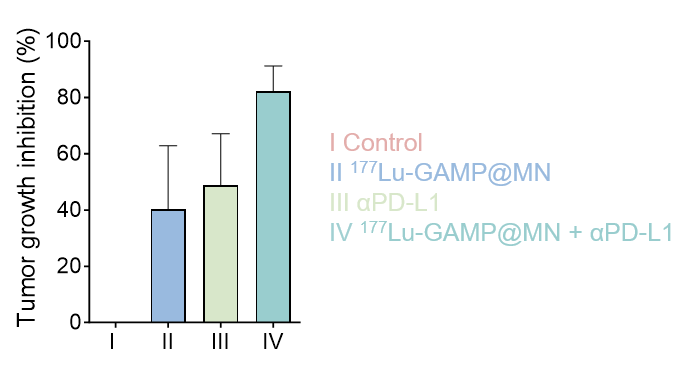


**Figure S28.** Tumor growth inhibition rate following different treatments.


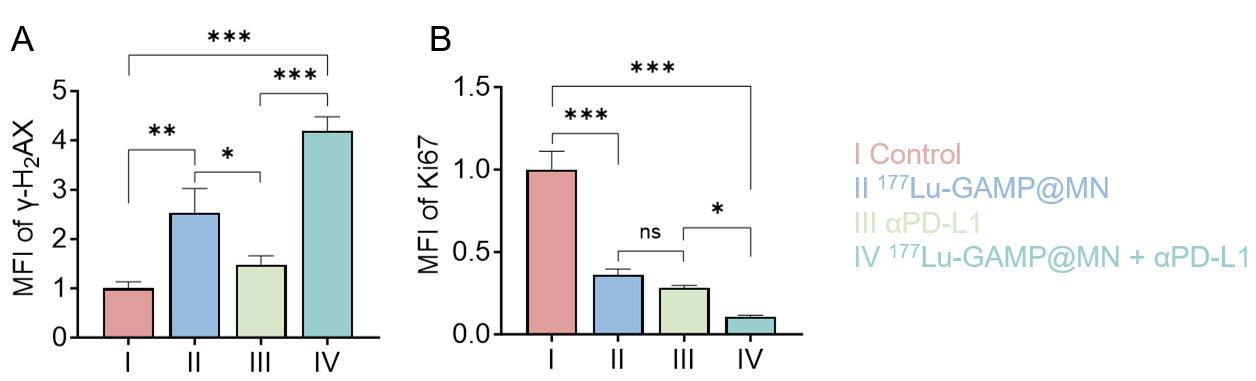


**Figure S29.** Quantitative analysis of (A) γ-H_2_AX and (B) Ki67 following various treatments.

**Table S1.** The hydrodynamic diameter, polydispersity index (PDI), and ζ potential of various nanoparticles.


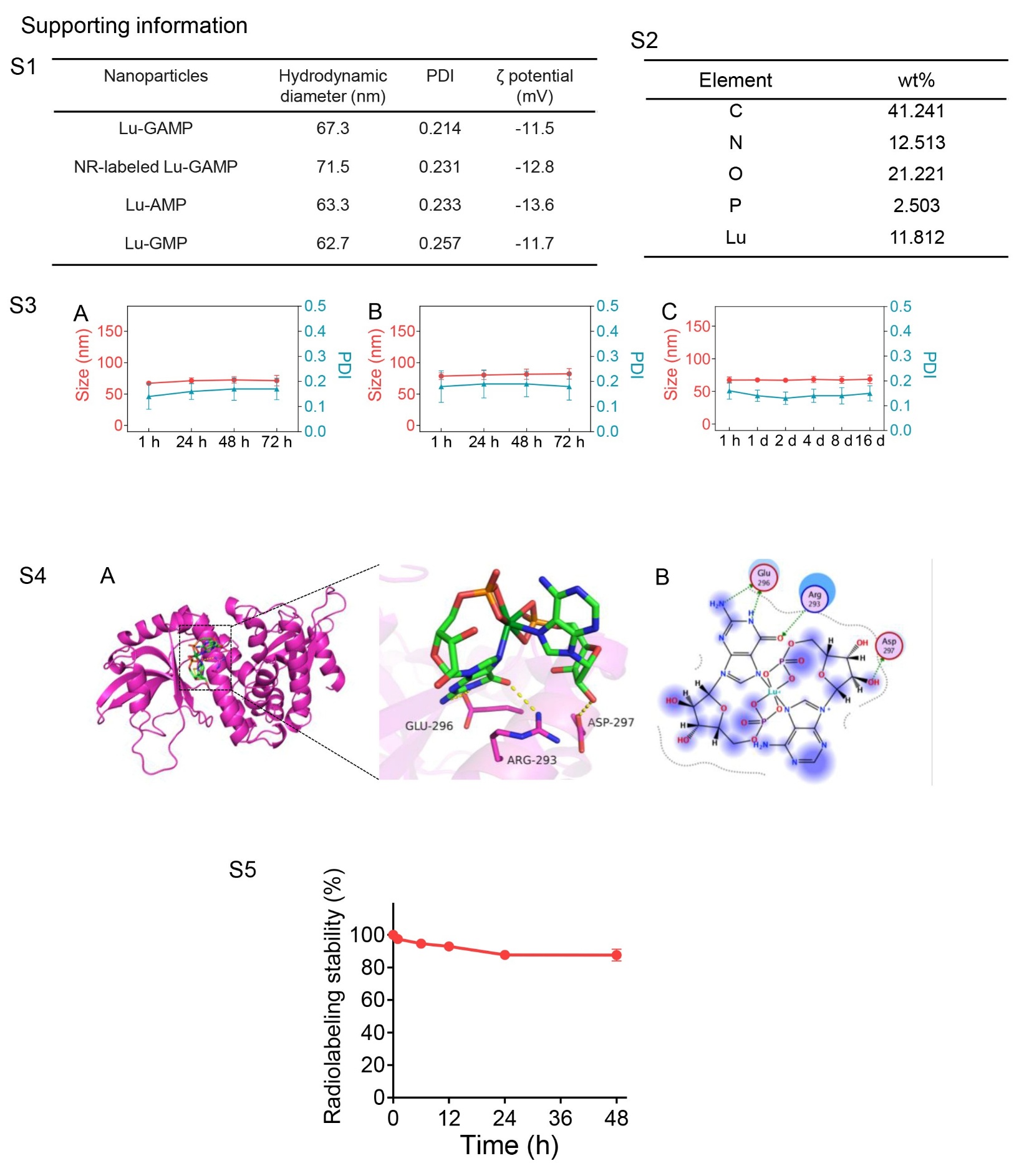


**Table S2.** The elemental composition of Lu-GAMP was analyzed using energy dispersive X-ray spectroscopy.


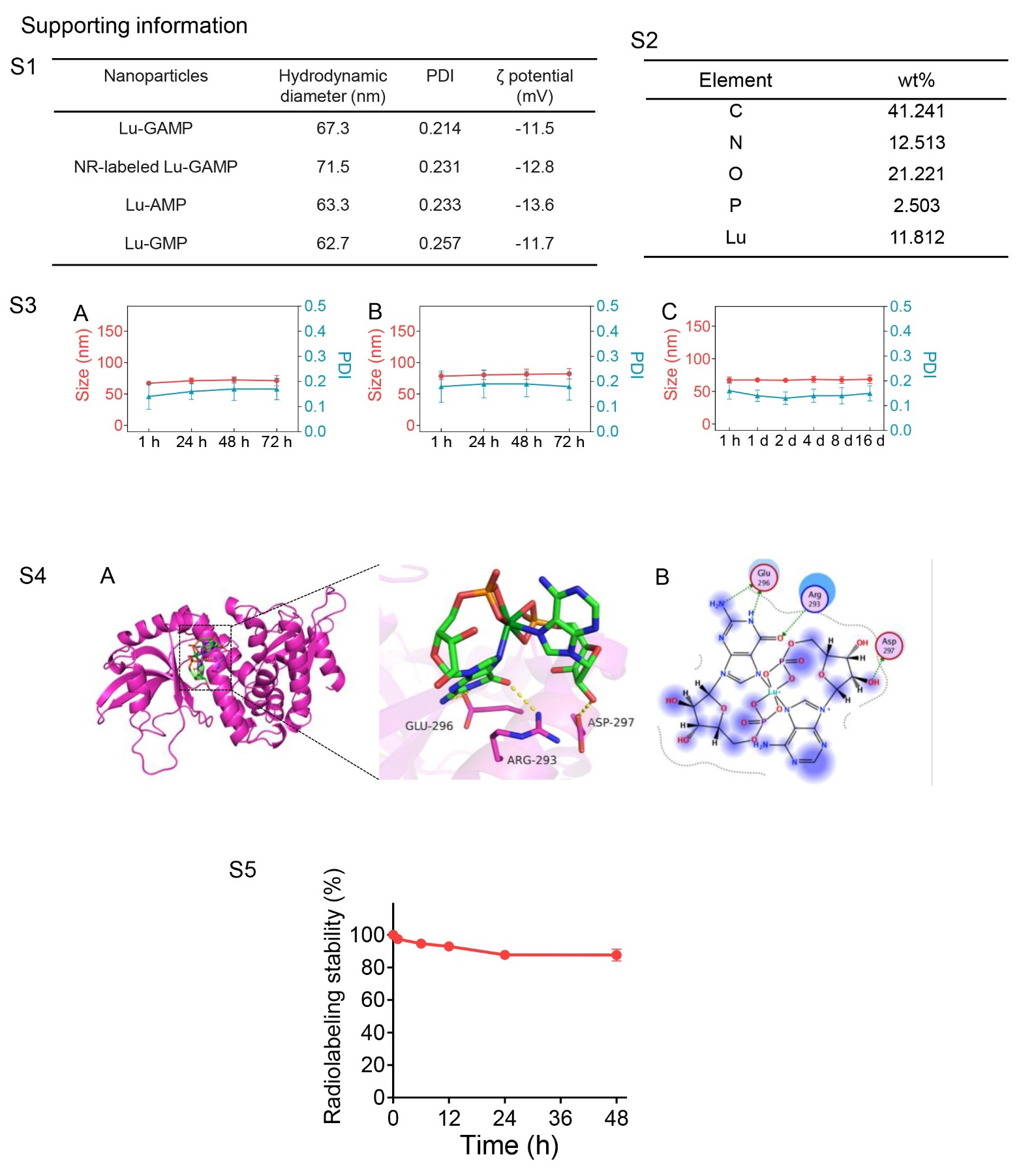


**Table S3.** Protein-ligand binding free energy components.


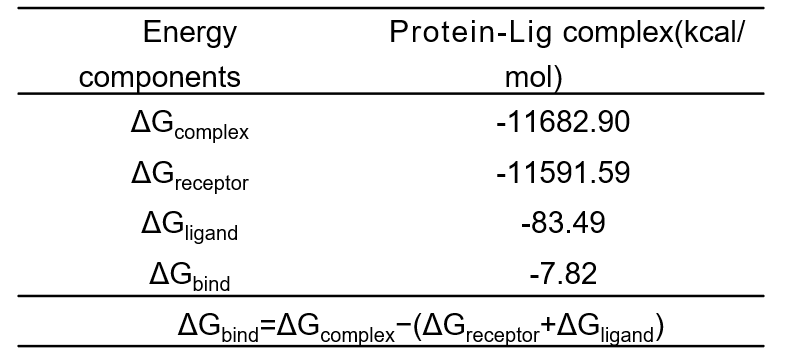


**Table S4.** Gamma counter analysis of ^177^Lu activity in major organs and tumor tissue.


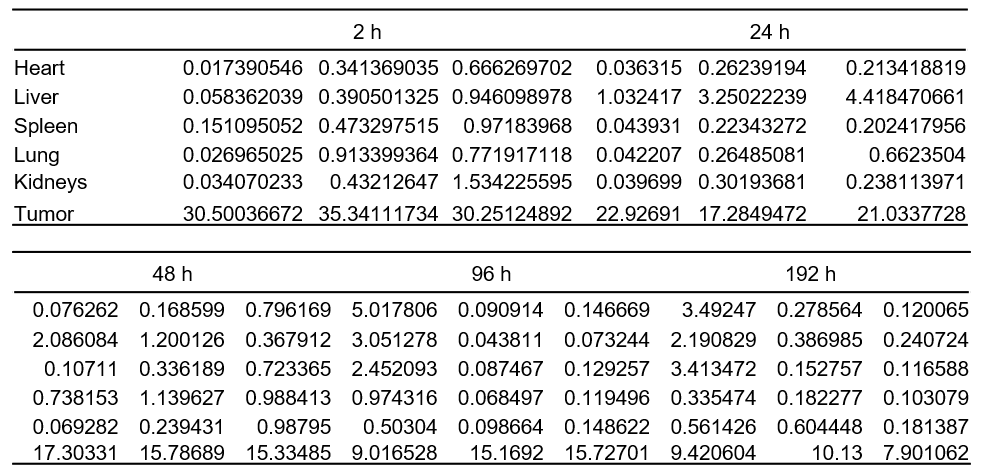


**Table S5.** Median overall survival in the primary tumor model.


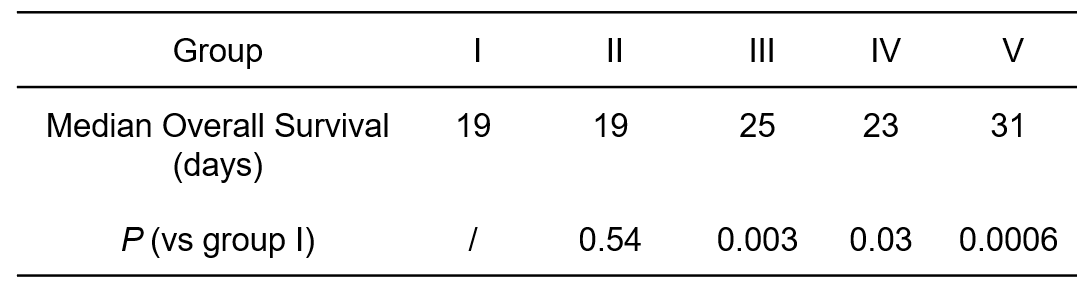


**Table S6.** Median overall survival in the distant tumor model.


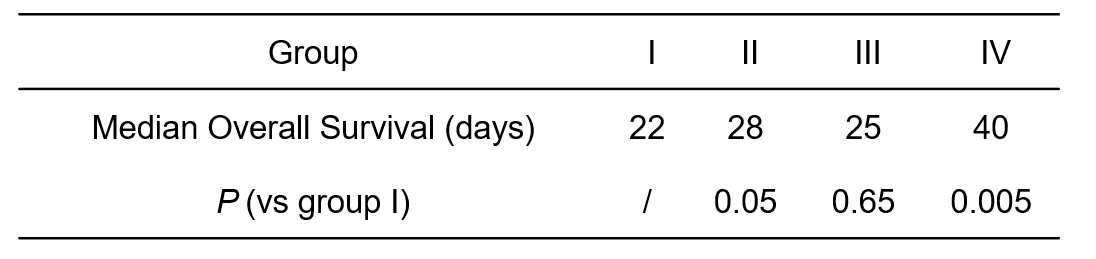

Supplement: Supplementary file 1 — Supporting File: exp270170‐sup‐0001‐SuppMat.docx. [file EXP2-6-20250737-s001.docx]
